# Graphene‐Engineered Rear Interfaces Enable Efficient Flexible CZTSSe Solar Cells on Metal Foils

**DOI:** 10.1002/advs.77181

**Published:** 2026-08-17

**Authors:** Yixiong Ji, Wentong Yang, Di Yan, Wei Luo, Jialu Li, Shi Tang, Jintao Fu, James Bullock, Mei Gao, Xin Li, Zhancheng Li, Jun Yang, Zhenghong Xiong, Yang Peng, Pengjun Zhao, Xingzhan Wei, Haofei Shi, Paul Mulvaney, Fangyang Liu

**Affiliations:** ^1^ State Key Laboratory of Functional Materials and Devices for Special Environmental Conditions Xinjiang Key Laboratory of Electronic Information Materials and Devices Xinjiang Technical Institute of Physics & Chemistry of CAS Urumqi China; ^2^ ARC Centre of Excellence in Exciton Science School of Chemistry University of Melbourne Victoria Australia; ^3^ School of Metallurgy and Environment Central South University Changsha China; ^4^ Department of Electrical and Electronic Engineering University of Melbourne Victoria Australia; ^5^ Chongqing Institute of Green and Intelligent Technology Chinese Academy of Sciences Chongqing China; ^6^ CSIRO Manufacturing Clayton Victoria Australia

**Keywords:** graphene, kesterite, ohmic contact

## Abstract

Flexible kesterite ${\mathrm{Cu}}_{2}\mathrm{ZnSn}$(S,Se)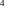 (CZTSSe) solar cells on metal foils are attractive for lightweight and conformable photovoltaic applications, yet their efficiencies remain significantly lower than those of rigid devices due to severe foil‐induced rear‐interface losses during high‐temperature chalcogenization. These effects include uncontrolled Mo(S,Se)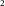 formation, interfacial instability, and the emergence of blocking back‐contact barriers that hinder efficient carrier extraction. Here, we introduce a single‐crystal graphene interlayer to engineer the Mo foil/CZTSSe interface. The atomically thin graphene layer decouples absorber growth from the mechanically and chemically non‐ideal foil surface, suppressing detrimental interfacial reactions and stabilizing the rear interface. As a result, carrier extraction is enhanced, and the back‐contact barrier is reduced toward quasi‐ohmic behavior. Flexible CZTSSe solar cells with graphene‐engineered interfaces achieve a champion efficiency of 11.6% with simultaneous improvements in $V_{\mathrm{OC}}$, $J_{\mathrm{SC}}$, and fill factor. These results highlight rear‐interface stabilization as a key strategy for improving flexible kesterite photovoltaics on metal substrates.

## Graphical abstract

An atomically thin graphene interlayer stabilizes the rear interface of flexible CZTSSe solar cells by regulating interfacial stress, suppressing detrimental interfacial reactions, and reducing the back‐contact barrier. This strategy simultaneously improves photovoltaic performance and mechanical durability.

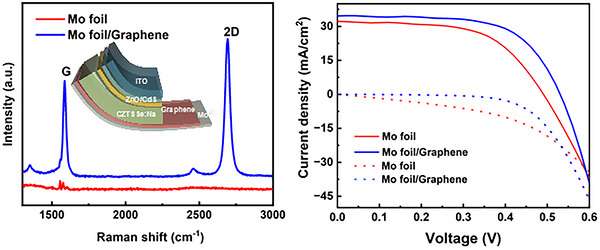

## 1 Introduction

Concerns over climate change driven by greenhouse‐gas emissions continue to accelerate the global transition toward carbon‐neutral energy systems [1]. Among renewable technologies, solar photovoltaics (PV) has emerged as a leading solution owing to its scalability and virtually inexhaustible energy supply. Beyond conventional rigid modules, flexible thin‐film PV is attracting increasing attention as a lightweight and conformable power source that can be deployed on curved or moving surfaces, enabling applications such as building‐integrated photovoltaics (BIPV), portable electronics, and emerging aerospace and wearable platforms [2].

Kesterite ${\mathrm{Cu}}_{2}\mathrm{ZnSn}$(S,Se)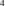 (CZTSSe) is a particularly promising absorber for flexible PV because it consists of earth‐abundant and relatively benign elements while remaining compatible with low‐cost thin‐film manufacturing. Over the past decade, substantial progress has been achieved in CZTSSe device engineering and efficiency improvement [3, 4, 5, 6]. Nevertheless, the performance of flexible CZTSSe solar cells still lags behind that of rigid devices fabricated on Mo‐coated soda‐lime glass, as well as several other flexible thin‐film technologies. This efficiency gap is generally attributed not only to the intrinsic $V_{OC}$ deficit of kesterite absorbers but also to additional materials and processing challenges introduced by flexible substrates.

For flexible CZTSSe devices fabricated on metal foils, two substrate‐related factors are frequently considered: (i) the absence of alkali elements typically supplied by soda‐lime glass, and (ii) the strong coupling between absorber formation and the foil back contact during high‐temperature chalcogenization [7, 8, 9]. While the alkali deficiency can be effectively compensated through alkali incorporation into precursors or post‐deposition treatments [6, 10, 11, 12], the second factor remains a major bottleneck for flexible architectures. In practice, raw Mo foils introduce more severe rear‐interface losses than those encountered in rigid devices. These include pronounced thermo‐mechanical deformation caused by thermal expansion mismatch, higher surface roughness that promotes interfacial inhomogeneity and local shunting pathways, and uncontrolled interfacial reactions that govern the formation of Mo(S,Se)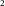 and the stability of the back contact [13, 14, 15, 16, 17, 18, 19]. Such effects can lead to void formation, nanoscale phase decomposition near the rear region, and the emergence of a blocking back‐contact barrier, collectively increasing series resistance and rear‐interface recombination [9, 19, 20, 21, 22]. Consequently, rear‐interface engineering has emerged as a key challenge for improving both efficiency and reliability in flexible CZTSSe solar cells.

Various interfacial modification strategies have been explored in rigid CZTSSe devices to suppress absorber decomposition and regulate the formation of Mo(S,Se)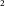. However, translating these approaches to flexible architectures remains challenging because the interlayers must simultaneously withstand high‐temperature processing, maintain adhesion on rough metal foils, and remain mechanically compliant under bending without introducing additional resistive losses. These constraints motivate the exploration of ultrathin interfacial modifiers that are thermally stable, chemically inert, electrically conductive, and mechanically flexible.

Herein, an ultrathin bilayer graphene interlayer is incorporated at the Mo foil/CZTSSe back contact. Its atomically thin and chemically inert nature provides a continuous template that partially decouples absorber growth from substrate roughness and interfacial chemical activity. As a result, the graphene interlayer improves the morphological uniformity of the bottom CZTSSe region, suppresses detrimental Mo–chalcogen reactions and nanoscale phase decomposition, and stabilizes the rear interface during high‐temperature processing. Electrical analysis further indicate that graphene enhances rear‐interface carrier extraction, as evidenced by improved long‐wavelength response, reduced leakage and series resistance, and a lowered back‐contact barrier approaching quasi‐ohmic behavior. By directly addressing the dominant rear‐interface bottleneck in flexible CZTSSe on metal foils, this graphene‐enabled interface engineering strategy provides a viable pathway toward efficient and mechanically robust kesterite photovoltaics.

## 2 Results and Discussion

**FIGURE 1:**
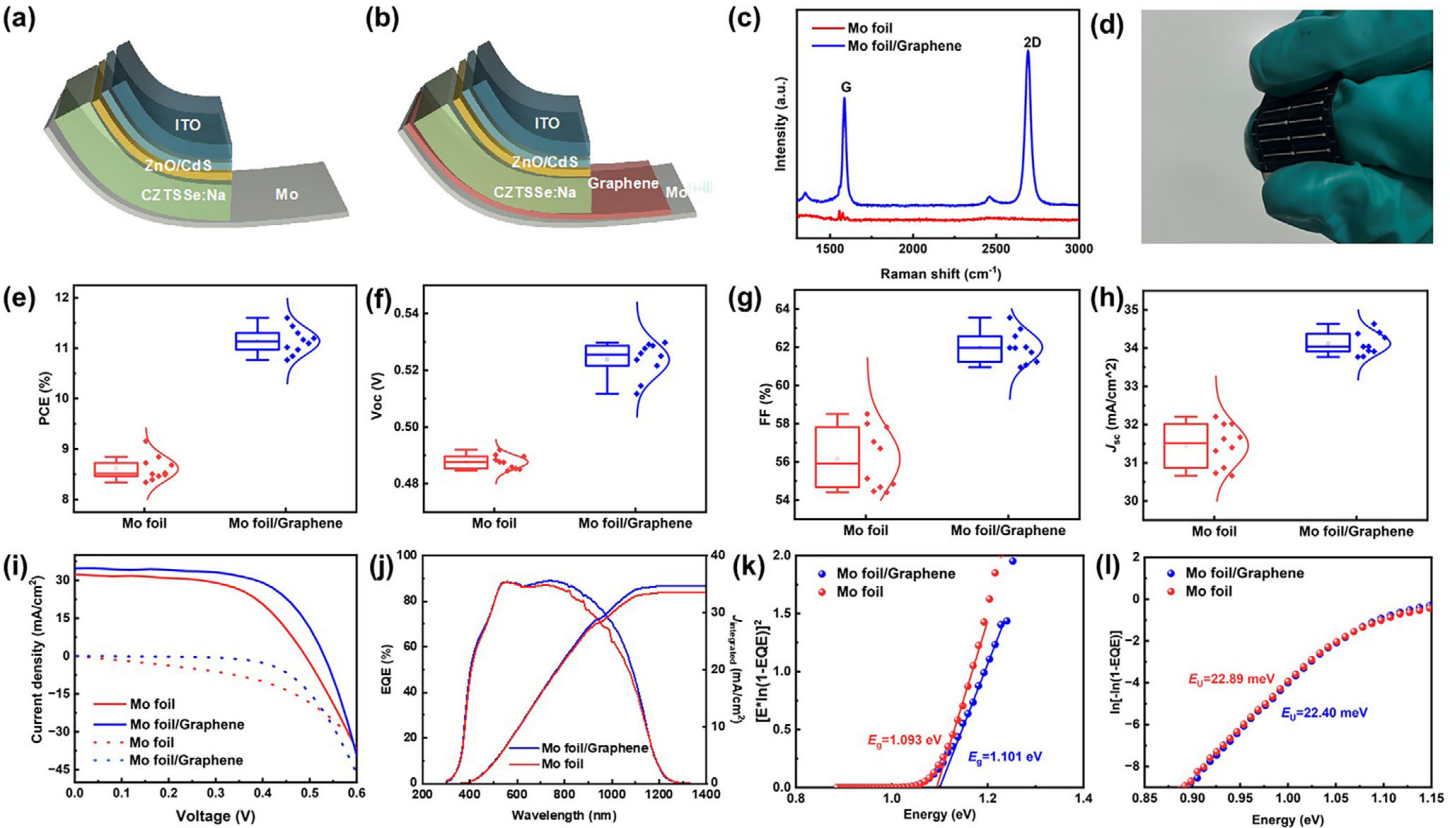
Flexible device structures without and with inserting graphene layers: (a) Mo foil/CZTSSe/CdS/ZnO/ITO (denoted as “Mo foil”), (b) Mo foil/Graphene/CZTSSe/CdS/ZnO/ITO (denoted as “Mo foil/Graphene”). (c) The corresponding Raman spectra of Mo foil and Mo foil/Graphene substrates were collected with 532 nm excitation. Photovoltaic properties of Mo foil and Mo foil/Graphene devices: Histogram of (e) $V_{OC}$, (f)$J_{SC}$, (g)FF, and (h)PCE. The photovoltaic statistics were obtained from 10 independently fabricated devices. (i) J‐V curves of the champion device and corresponding (j) EQE, (k) calculated Eg, and (l) calculated Urbach energy.

The device architecture of the flexible CZTSSe solar cells investigated in this work is illustrated in Figure 1, consisting of a Mo foil/Graphene/CZTSSe/CdS/ZnO/ITO structure. The graphene layer was transferred onto the Mo foil using a standard wet‐transfer process [23]. Raman spectroscopy (Figure 1c) reveals a weak D peak at $∼1350\;{\mathrm{cm}}^{-1}$, a strong G peak at $∼1580\;{\mathrm{cm}}^{-1}$, and a pronounced 2D peak at $∼2700\;{\mathrm{cm}}^{-1}$. The low $I_{D}/I_{G}$ ratio of 0.23 indicates the minimal defect density introduced during the transfer process, while the $I_{2D}/I_{G}$ ratio of 1.38 (within the range of 1–2) confirms the high crystalline quality of the transferred graphene films [24].

To isolate the influence of the rear‐interface modification, the potential impact of alkali deficiency in flexible substrates was minimized by incorporating Na directly into the CZTS precursor solution, a strategy previously demonstrated to enable high‐performance CZTSSe devices on rigid substrates [6, 12, 25]. Therefore, in the following discussion, the performance differences between the flexible devices can be primarily attributed to foil‐induced rear‐interface effects and their impact on carrier extraction and recombination dynamics.

Figure 1e–h summarizes the photovoltaic statistics of the flexible CZTSSe devices fabricated on raw Mo foil and Mo foil/Graphene substrates. Compared with the control devices, the graphene‐modified devices exhibit simultaneous improvements in $V_{OC}$, $J_{SC}$, and fill factor, leading to a substantially higher power conversion efficiency. As summarized in Table 1, the champion graphene device reaches a PCE of 11.6%, significantly exceeding the 9.16% efficiency obtained for the Mo foil control. These improvements support the central hypothesis proposed in the Introduction: the dominant performance limitation in flexible CZTSSe devices on raw metal foils originates from an unstable and nonuniform rear interface, which introduces recombination losses and resistive penalties. The graphene interlayer mitigates these effects by stabilizing the back interface and facilitating more efficient carrier extraction. Additionally, Figure S1 presents the photovoltaic parameters of 30 devices fabricated on $4\times 4\;{\mathrm{cm}}^{2}$ graphene‐coated Mo foils, demonstrating good device uniformity and process reproducibility. Mechanical robustness was evaluated by outward bending at a radius of 6 mm for 1000 cycles (Figure S3). Figure S2 shows the normalized PCE as a function of bending cycles, and the corresponding photovoltaic parameters are summarized in Tables S1 and S2. The graphene‐based devices exhibit significantly improved bending stability and mechanical durability.

**TABLE 1:** The photovoltaic and diode parameters of the best Mo foil and Mo foil/Graphene solar cells. The values of $E_{g}$ are obtained from the EQE data. The diode parameters are extracted from dark J–V curves.

Device	$V_{OC}$ (V)	$J_{SC}$ (mA/${\mathrm{cm}}^{2}$)	FF (%)	PCE (%)	A
Mo foil	0.49	32.21	57.99	9.16	2.61
Mo foil/G	0.53	34.63	62.97	11.60	1.43
Device	$J_{0}$ (mA/${\mathrm{cm}}^{2}$)	$R_{S}$ (ohm ${\mathrm{cm}}^{2}$)	$G_{SH}$ (mS/${\mathrm{cm}}^{2}$)	Eg/q‐$V_{OC}$ (eV)	$E_{U}$ (meV)
Mo foil	$9.12\times 10^{-4}$	1.47	0.18	0.60	22.89
Mo foil/G	$4.15\times 10^{-5}$	0.50	0.06	0.57	22.40

Further insights into the device operation can be obtained from the extracted diode parameters listed in Table 1. The related calculations are shown in Figure S10. The graphene device exhibits a substantially reduced series resistance $R_{s}$ and shunt conductance $G_{sh}$, which directly explain the improved fill factor and reduced leakage pathways often associated with rough metal foils. Meanwhile, the dark saturation current density $J_{0}$ decreases by nearly an order of magnitude, indicating suppressed non‐radiative recombination. In contrast, the extracted Urbach energies remain nearly unchanged between the two devices, suggesting that the band‐tail distribution and intrinsic bulk disorder of the CZTSSe absorber are largely unaffected. These results indicate that graphene does not primarily modify the bulk electronic structure of CZTSSe; rather, it mitigates foil‐induced losses associated with rear‐interface instability, interfacial secondary phases, and contact barriers.

The current–voltage characteristics provide further evidence for improved back‐contact behavior. As shown in Figure 1i, the Mo foil control device exhibits a pronounced J–V crossover behavior, which is commonly associated with non‐ideal rear contacts or barrier‐type interfaces [26]. In contrast, the graphene‐modified device displays a more regular diode response with reduced distortion in the forward‐bias region, consistent with a lower series resistance and improved back‐contact selectivity. The external quantum efficiency (EQE) spectra in Figure 1j further reveal an enhanced photoresponse in the long‐wavelength region for the Mo foil/Graphene device. Because long‐wavelength photons are absorbed deeper in the CZTSSe absorber, the corresponding carriers must travel toward the rear interface before collection. Therefore, the improved long‐wavelength EQE indicates more efficient carrier extraction from the bottom absorber region. Combined with the nearly identical Urbach energies (Figure 1l) and bulk‐defect concentrations (Figure S5), these results suggest that the performance enhancement mainly originates from improved carrier collection near the back contact rather than from changes in the intrinsic bulk properties of the absorber [27].

The origin of this improvement can be understood by considering the flexible‐specific reaction environment during high‐temperature selenization. Compared with rigid Mo/glass substrates, Mo foils experience larger thermo‐mechanical deformation and provide more diffusion pathways for Se (Figure S4), which can accelerate interfacial reactions and destabilize the rear interface [28]. In such cases, excessive interfacial reaction may trigger a cascade of degradation processes, including void formation (Figure S7), phase decomposition, insufficient grain growth (Figure S8), and the development of blocking back‐contact barriers. The atomically thin graphene interlayer mitigates the coupling between thermomechanical deformation and interfacial reactions. Thermal‐expansion calculations (Figure S4) show that, at the same interfacial Mo(S,Se)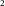 thickness, graphene suppresses the expansion associated with Mo(S,Se)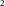 formation, thereby stabilizing the rear interface and promoting uniform CZTSSe growth. In contrast, the ${\mathrm{Al}}_{2}O_{3}$ control exhibits weaker XRD and Raman signals (Figure S8), indicating poorer CZTSSe crystallinity due to less effective stress relaxation. The superior photovoltaic performance of the graphene device (Figure S9) further demonstrates that graphene uniquely combines interfacial stress regulation with efficient charge transport, rather than acting merely as a passive interlayer.

**FIGURE 2:**
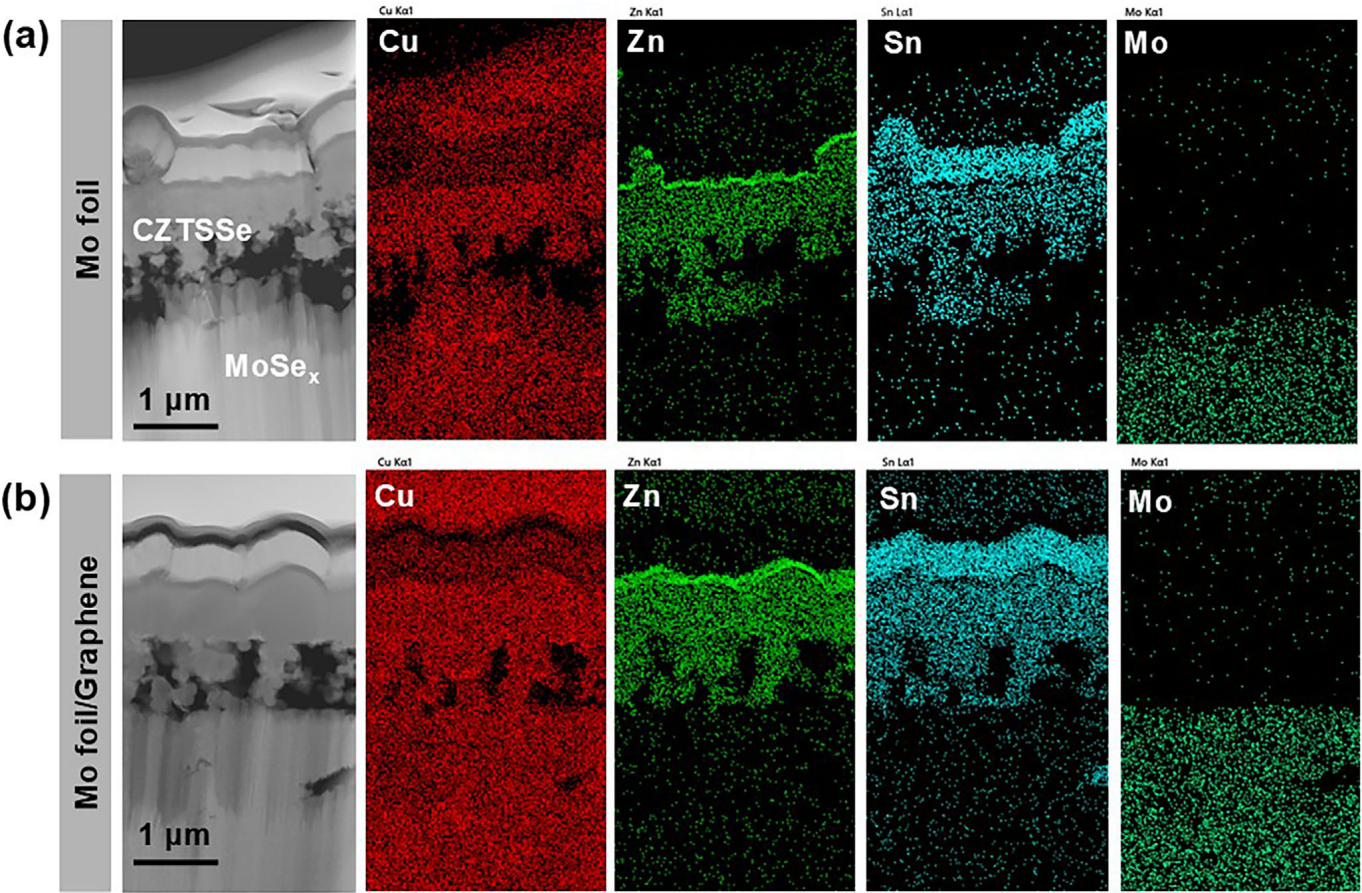
Cross‐sectional TEM and EDS mappings of the elemental composition distribution of the flexible CZTSSe solar cells: (a) Mo foil device, (b) Mo foil/Graphene device.

Cross‐sectional TEM and EDS mapping were employed to directly examine the microstructural differences between the two devices (Figure 2). The Mo foil control device exhibits a highly nonuniform Mo(S,Se)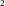/CZTSSe interface accompanied by void formation and local structural collapse in the bottom region. Such structural irregularities can lead to current crowding, shunting pathways, and enhanced recombination at the rear interface, consistent with the degraded electrical characteristics observed in the control device. In addition, uneven attachment of CZTSSe grains to the Mo(S,Se)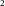 layer suggests that the rough and chemically heterogeneous foil surface can induce nonuniform nucleation and facilitate local phase separation during growth. In contrast, the Mo foil/Graphene device exhibits a smoother and more continuous rear heterointerface. The presence of graphene effectively passivates reactive sites on the Mo surface and provides a more uniform growth template for CZTSSe nucleation. As a result, the bottom absorber region shows improved structural uniformity and reduced interfacial defects. These microstructural improvements are consistent with the enhanced long‐wavelength EQE response and the reduced $R_{s}$ and $G_{sh}$ extracted from electrical measurements.

**FIGURE 3:**
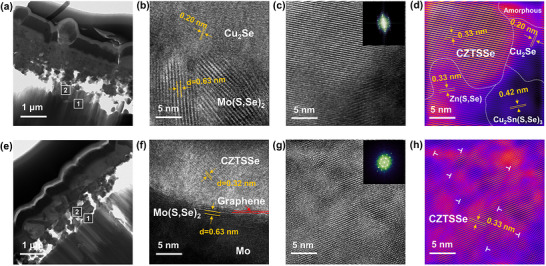
Cross‐sectional TEM images in bright field configuration of (a) Mo foil device and (e) Mo foil/Graphene device. HRTEM images of (b) region 1 and (c) region 2 in the Mo foil device, and (f) region 1 and (g) region 2 in the Mo foil/Graphene device. The insets in (c) and (g) are the corresponding FFT images. (d) and (h) are IFFT images of region 2 of two devices, respectively.

High‐resolution TEM (HRTEM) further reveals the atomistic characteristics of the rear interface (Figure 3). In the Mo foil control device, the Mo(S,Se)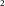 layer exhibits vertical growth accompanied by ${\mathrm{Cu}}_{2}\mathrm{Se}$ deposition, indicating a non‐equilibrium reaction environment during selenization. Such vertically textured and chemically heterogeneous interfaces can significantly deteriorate rear‐contact selectivity and introduce recombination‐active defects. By contrast, the Mo foil/Graphene device exhibits a more parallel Mo(S,Se)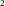 orientation and improved crystallinity in the adjacent CZTSSe region. This configuration is expected to be less detrimental to vertical carrier transport and indicates a more stable interfacial growth environment [29, 30].

In addition, regions slightly away from the interface (Figure 3c,g) reveal clear differences in phase purity. The Mo foil control device exhibits detectable secondary phases, whereas the graphene device maintains a predominantly single‐phase CZTSSe structure with larger grains in the bottom region. Furthermore, this can be confirmed from the IFFT images of region II in both devices that numerous secondary‐phase regions in the control device, whereas the graphene device contains mainly single‐phase CZTSSe with only a few localized dislocations. These observations suggest that graphene stabilizes the local reaction environment during absorber formation, suppressing foil‐induced decomposition and promoting more uniform grain growth.

**FIGURE 4:**
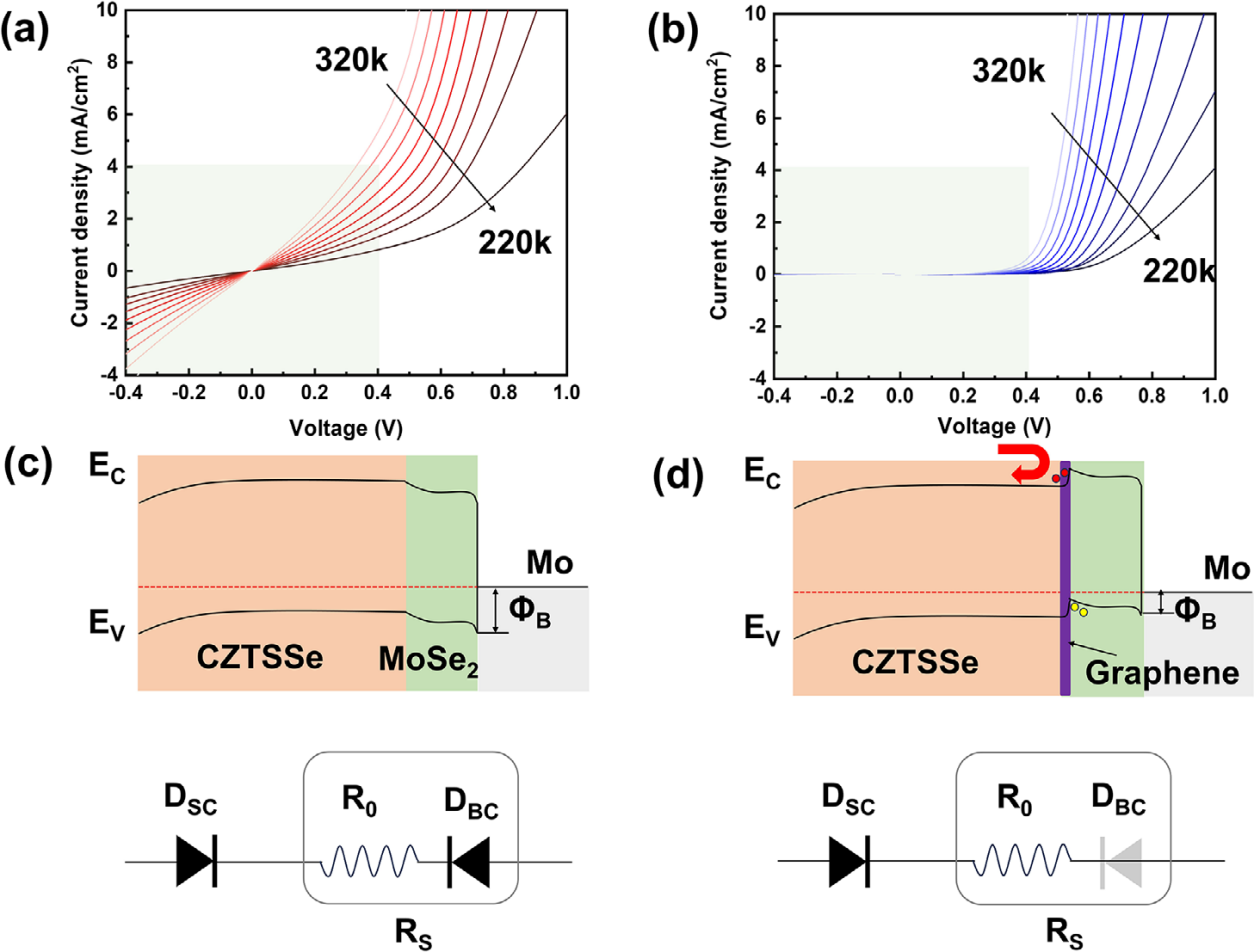
Temperature‐dependent dark J–V curves for Mo foil (a) and Mo foil/Graphene (b) devices. Hypothetical back contact diagrams and circuit models are shown in (c) and (d). $D_{SC}$ is the solar cell diode. $R_{S}$ is series resistance, consisting of background or residual series resistance ($R_{0}$) and a blocking back contact diode $D_{BC}$.

To directly probe the electrical properties of the rear contact, temperature‐dependent dark J–V measurements were performed (Figure 4). For both devices, the extracted series resistance increases as temperature decreases, indicating thermally activated carrier transport across the rear interface. Series resistance of the two devices is extracted and shown in Figure S11, by following the Equation (1), where n is the diode ideality factor, $G_{S}$ is shunt resistance, T is the temperature, q is the electron charge, and k is the Boltzmann constant, respectively. However, this temperature dependence is significantly stronger in the Mo foil control device, suggesting the presence of a more pronounced blocking barrier at the Mo/CZTSSe interface.

(1) $$\;dV/dJ=R_{S}+nkT/q\left(J-G_{S}V\right)$$

(2) $$\;R_{\mathrm{BC}}=\frac{k}{qA^{*}T}\exp\left(\Phi_{B}/kT\right)$$

The temperature‐dependent data were fitted using the equivalent‐circuit model shown in Figure 4c,d, which consists of a main solar‐cell diode ($D_{\mathrm{SC}}$), a back‐contact diode ($D_{\mathrm{BC}}$), and a background series resistance ($R_{0}$) accounting for the top layers and the bulk resistance of the CZTSSe absorber. Under forward bias, the back‐contact diode is reverse‐biased, and its current is limited by the reverse saturation current. As the temperature decreases, this current rapidly decreases, leading to an increase in the apparent series resistance (Figure S11). Based on this model, the back‐contact barrier height was extracted using Equation (2), where $A^{*}$ is the effective Richardson constant and $\Phi_{B}$ is the barrier height. The extracted $\Phi_{B}$ decreases from approximately 0.42 eV for the Mo‐foil device to 0.32 eV for the graphene‐modified device. This reduction is consistent with graphene‐induced modulation of the back‐interface band alignment, which weakens the reverse‐junction barrier and shifts the contact toward quasi‐ohmic behavior. Consequently, photogenerated holes are transported more readily toward the Mo back electrode, while electrons are directed toward the CdS layer, thereby reducing back‐contact transport losses and interfacial recombination.

**FIGURE 5:**
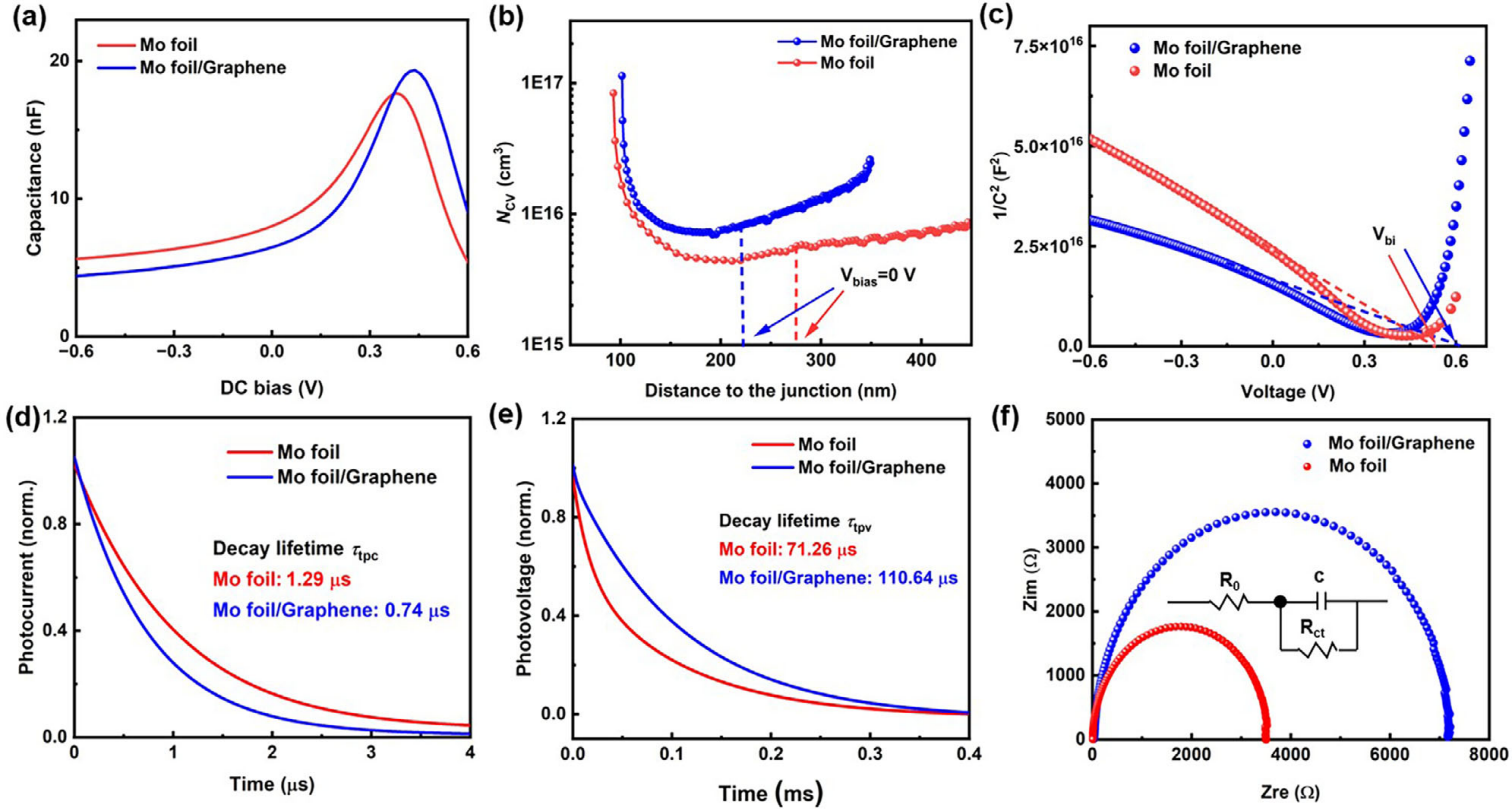
(a) C–V curves. (b) CV curves and the calculated width of the space charge region are shown as the dashed lines at 0 V bias. (c) Plot of 1/C2 vs. voltage showing the variation in the built‐in potential, $V_{bi}$. Normalized TPC spectra in (d) and normalized TPV spectra in (e). (f) Impedance spectra for the Mo foil and Mo foil/Graphene devices.

Capacitance–voltage (C–V) analysis further provides insight into the junction properties of the devices (Figure 5). The graphene device exhibits a higher apparent carrier density and a reduced depletion width, suggesting improved electrical conductivity in the absorber/contact region. Meanwhile, the built‐in potential increases from ∼0.51 V to ∼0.61 V, providing a direct electrical explanation for the improved $V_{OC}$.

Carrier dynamics measurements were performed using transient photocurrent (TPC) and transient photovoltage (TPV) techniques, which also reflected the optimized band alignment at the rear interface induced by inserting graphene. As shown in Figure 5, the graphene device exhibits a significantly longer recombination lifetime ($\tau_{TPV}$) compared with the control device, indicating suppressed carrier recombination. At the same time, the carrier transport lifetime ($\tau_{TPC}$) becomes shorter, suggesting faster carrier extraction. When interpreted together with the structural observations, these results indicate that graphene suppresses foil‐induced interfacial heterogeneity and recombination centers while simultaneously facilitating more efficient charge transport.

Electrochemical impedance spectroscopy (EIS) measurements further confirm this interpretation. As shown in Figure 5f, the graphene device exhibits a substantially larger recombination resistance $R_{rec}$ than the Mo foil control device, indicating suppressed recombination losses. Together with the longer TPV lifetime and improved transport dynamics, these results demonstrate that graphene significantly improves the carrier extraction and recombination behavior of flexible CZTSSe devices. Additionally, admittance spectroscopy (AS) spectra (Figure S5) reveal only a slight reduction in defect‐state density for the graphene device, suggesting that the performance enhancement is not mainly caused by bulk defect suppression, but is more closely related to improved rear‐interface properties and charge transport.[Table advs77181-tbl-0002]

**TABLE 2 advs77181-tbl-0002:** Summary of the results derived from C‐V, TPV, TPC, and EIS measurements.

Device	$V_{bi}$ (V)	$N_{CV}$ (${\mathrm{cm}}^{-3}$)	Depletion width (nm)	$\tau_{tpv}$ (μs)	$\tau_{tpc}$ (μs)	$R_{rec}$ (kΩ)
Mo foil	0.51	$5.68\times 10^{15}$	275.75	71.26	1.29	3.53
Mo foil/G	0.61	$8.21\times 10^{15}$	222.17	110.64	0.74	7.17

Overall, the combined structural and electrical evidence supports a consistent mechanism: the graphene interlayer stabilizes the Mo foil/CZTSSe rear interface during high‐temperature chalcogenization, promoting a parallel Mo(S,Se)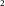 orientation at the interface, reducing the interfacial decomposition and structural inhomogeneity. This stabilization enables a more uniform bottom absorber region and reduces the back‐contact barrier, allowing the rear interface to approach quasi‐ohmic behavior. As a result, carrier extraction is enhanced and recombination losses are suppressed, leading to simultaneous improvements in $V_{OC}$, $J_{SC}$, and fill factor.

### 2.1 Conclusion

In summary, this work identifies rear‐interface instability as a major limitation for flexible CZTSSe solar cells fabricated on metal foils. During high‐temperature chalcogenization, raw Mo foils tend to induce severe back‐interface degradation, including excessive Mo(S,Se)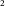 formation, interfacial voids, and local phase decomposition, which lead to increased recombination and the formation of blocking back‐contact barriers. By introducing a graphene interlayer between the Mo foil and the CZTSSe absorber, the rear interface can be effectively stabilized. Structural characterization reveals a smoother and more uniform Mo(S,Se)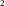/CZTSSe interface with suppressed interfacial defects in the bottom absorber region. Electrical analyzes further demonstrate that graphene primarily improves the back‐contact properties, reducing the back‐contact barrier height and enabling quasi‐ohmic carrier extraction. As a result, the graphene‐engineered flexible CZTSSe devices exhibit simultaneous improvements in $V_{\mathrm{OC}}$, $J_{\mathrm{SC}}$, and FF, achieving a champion efficiency of 11.6%. These results highlight the importance of rear‐interface engineering in flexible kesterite photovoltaics and demonstrate that graphene‐enabled interface decoupling offers an effective strategy for improving back‐contact quality in CZTSSe devices on metal foils.

## 3 Experimental Procedures

### 3.1 Reagent and Materials

The following chemicals were used without further refining: CuCl (99.999%, Macklin), Zn(${\mathrm{CH}}_{3}\mathrm{COO}$)
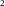
2$H_{2}O$ (99.995%, Aladdin), ${\mathrm{SnCl}}_{4}$5$H_{2}O$ (99.995%, Aladdin), NaCl (99.99%, Aladdin), thiourea (99%, Aladdin), ${\mathrm{CdSO}}_{4}$8/3$H_{2}O$ (99%, Aladdin), 2‐methoxyethanol (99.9%, Aladdin), ${\mathrm{NH}}_{3}H_{2}O$ (25%, Aladdin), Se (99.999%, ZhongNuo). Single‐crystal graphene was grown by Zhancheng Li and Xin Li from CIGIT. Single‐crystal monolayer graphene was transferred onto the Mo foil by the commonly used PMMA‐assisted wet‐transfer method [31]. ${\mathrm{Al}}_{2}O_{3}$ was deposited from ALD. Mo foil substrates were purchased from Shangyang Co., Ltd. in China.

### 3.2 CZTSSe Absorber

The CZTS precursor solution was fabricated by dissolving CuCl (0.3743g), Zn(${\mathrm{CH}}_{3}\mathrm{COO}$)
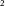
2$H_{2}O$ (0.6190 g), ${\mathrm{SnCl}}_{4}$5$H_{2}O$ (0.8940g), NaCl (0.0293 g), and thiourea (1.5186 g) into 2‐methoxyethanol(10 ml). The solution was kept stirred until the color of the final solution was pale yellow. The precursor solution was spin‐coated onto Mo foil at 3000 rpm for 40 s, followed by baking at 285 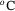 for 2 min. The above steps were repeated several times to fabricate 1.5 μm precursor films. Then, a selenization process at $550^{o}C$ for 15 min in a rapid thermal process (RTP) furnace was used to form the final CZTSSe absorbers [28].

### 3.3 Device Fabrication

The architectures of all samples are listed below: Mo foil device: Mo foil/CZTSSe/CdS/ZnO (60 nm)/ITO/Ag. Mo foil/Graphene or Mo/foil/G device: Mo foil/Graphene/CZTSSe/CdS/ZnO (60 nm)/ITO/Ag. Mo foil/${\mathrm{Al}}_{2}O_{3}$ device: Mo foil/${\mathrm{Al}}_{2}O_{3}$/CZTSSe/CdS/ZnO (60 nm)/ITO/Ag.

The selenized films were immersed into a solution mixed with 150 ml DI water, 20 mL 0.015 mol $L^{-1}$ ${\mathrm{CdSO}}_{4}$ solution, 20 mL 0.75 mol $L^{-1}$ thiourea solution, and 25 mL ammonium hydroxide at 75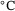 to complete the deposition of the 50 nm CdS buffer layer. Next, 60 nm i‐ZnO and 300 nm ITO were sequentially magnetron‐sputtered on the top of the buffer layer to complete the fabrication of the device. Finally, 300 nm Ag grids were thermally evaporated as a top electrode. No antireflection layer was used.

### 3.4 Characterization

Top‐view and cross‐sectional images were collected using a JEOL JSM‐7900F field emission scanning electron microscope (FESEM). The acceleration voltage was 5 kV. To verify structural properties and phase purity, X‐ray diffraction (XRD) and Raman measurements were conducted on PANalytical Empyrean 2 and inVia Renishaw (532 nm excitation wavelength) instruments respectively. The reported photovoltaic parameters were determined based on the total device area ($0.2345\;{\mathrm{cm}}^{2}$). All current density–voltage (J–V) measurements were performed in the forward‐scan direction under simulated AM 1.5G illumination ($100\;\mathrm{mW}\;{\mathrm{cm}}^{-2}$) using a Keithley 2400 SourceMeter. The J‐V characteristics of CZTSSe devices were measured using a Xenon‐lamp‐based solar simulator (Newport, AM 1.5G, 100 mW ${\mathrm{cm}}^{-2}$) in conjunction with a Keithley 2400 SourceMeter. The external quantum efficiencies (EQE) were measured using a QE‐R measurement system (Enli Tech) and were calibrated using Enli Tech. Lab‐certified Si and Ge reference photodiodes. The capacitance–voltage (C–V) measurements were performed with an impedance analyzer at a frequency of 100 kHz with a DC bias voltage sweeping from –1.0 to 0.6 V. Drive‐level capacitance profiling (DLCP) measurements were carried out using AC amplitudes varying from 40 mV to 140 mV and with DC bias voltages that swept from –1.0 to 0.8 V. Transient photocurrent (TPC) measurements were performed using a semiconductor characterization system (Agilent B1500A). The illumination was supplied by a monochromatic light‐emitting diode (LED), which was controlled by an Agilent 33210A function generator.

## 4 Statistical Analysis

The statistical data for the CZTSSe solar cells were analyzed using Origin 2024. Each statistical analysis included 10 or 30 devices. Device parameters, including $V_{\mathrm{OC}}$, $J_{\mathrm{SC}}$, FF, and PCE, are presented as box plots showing the median, mean, interquartile range, and outliers. Data normality and distribution were also assessed.

## Conflicts of Interest

The authors declare no conflicts of interest.

## Supporting information

Supporting file

## Data Availability

The data that support the findings of this study are available on request from the corresponding author. The data are not publicly available due to privacy or ethical restrictions.
