# Supplementary material for: Graphene‐Engineered Rear Interfaces Enable Efficient Flexible CZTSSe Solar Cells on Metal Foils
Source: Adv Sci (Weinh). 2026 Aug 17;13(62):e77181. doi: 10.1002/advs.77181 (PMC13479858; doi:10.1002/advs.77181)
Supplement: Supplementary file 1 [file ADVS-13-e77181-s001.pdf]

# Supporting Information Available

## Bending Test of flexible cells

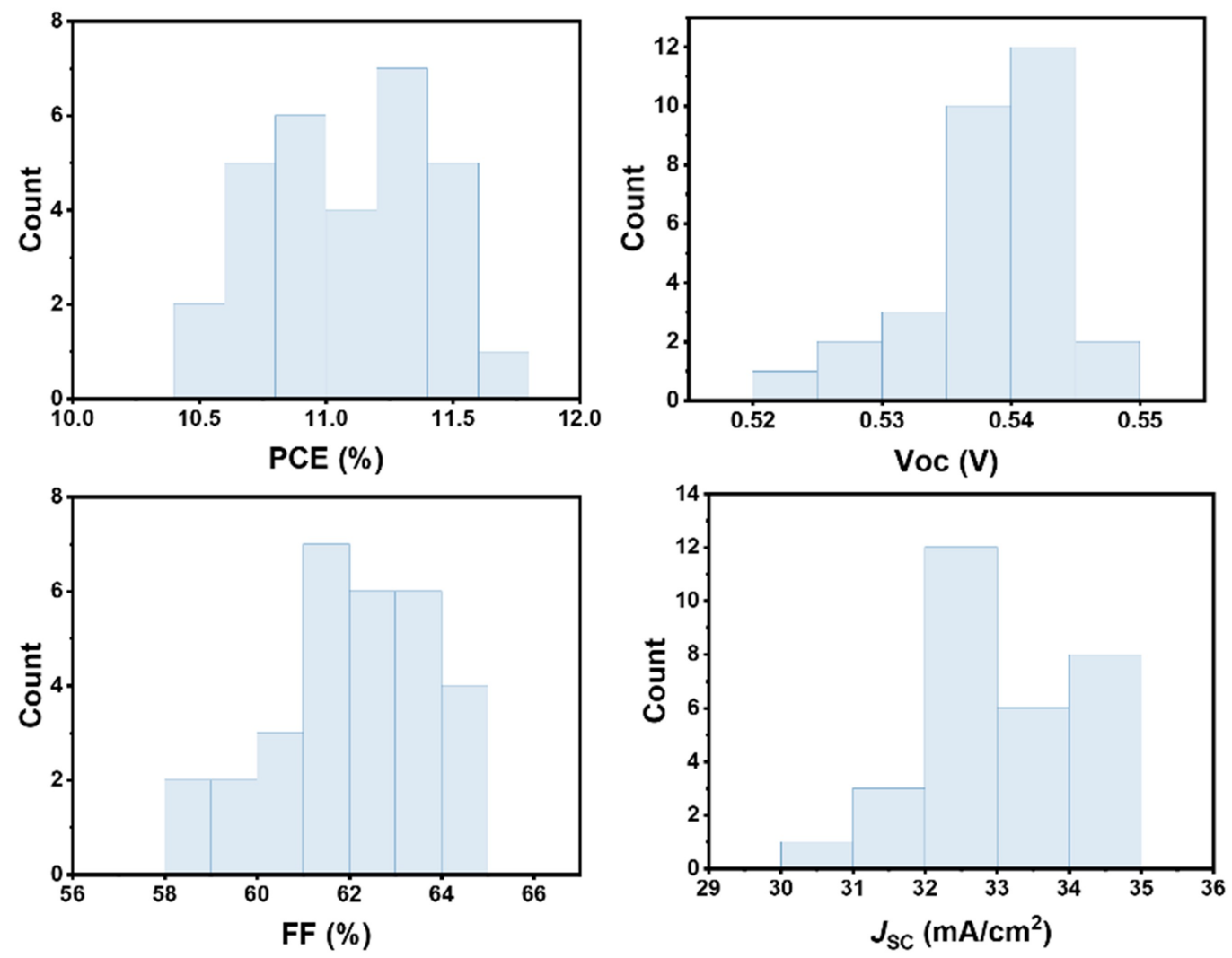


Figure S1: The photovoltaic properties distribution of Mo foil/Graphene devices.

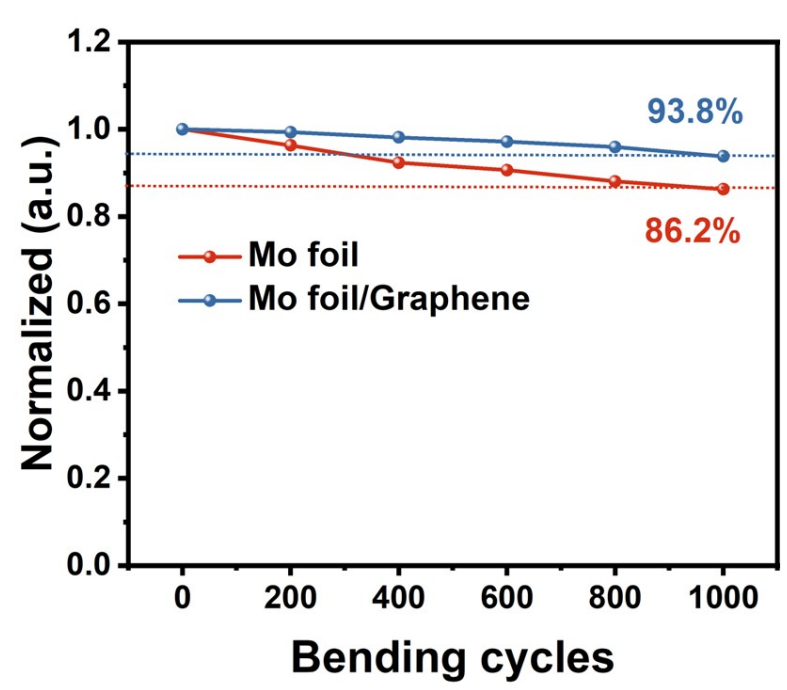


Figure S2: The normalized PCEs of CZTSSe devices as a function of bending cycles.

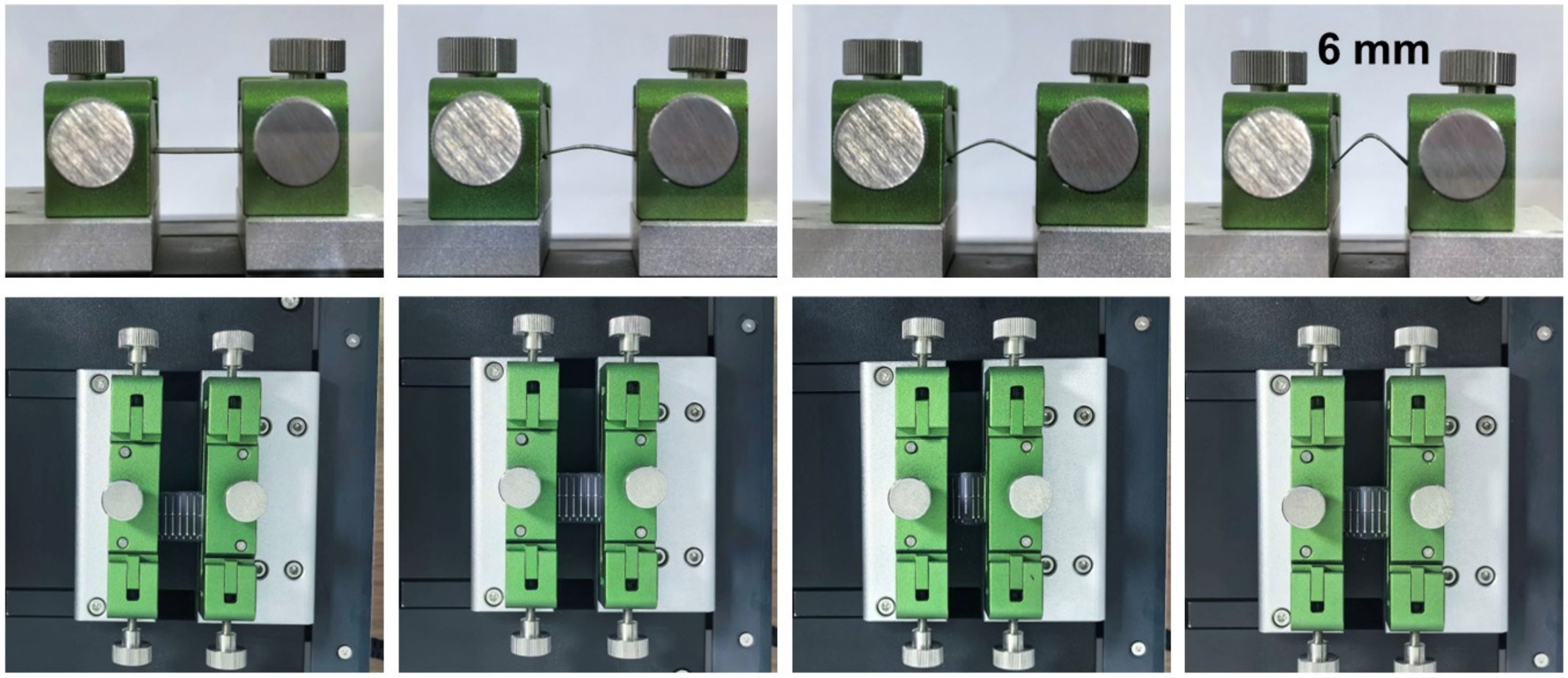


Figure S3: Bending test of flexible CZTSSe solar cells.

Table S1: Photovoltaic parameters of the Mo CZTSSe device after different bending cycles.

| **Bending cycles (cycles)** | $V_{OC}$ **(V)** | **PCE (%)** | **FF (%)** | $J_{SC}$ **(mA cm$^{-2}$)** |
|---|---|---|---|---|
| 0 | 0.48666 | 9.10489 | 60.01336 | 31.17436 |
| 200 | 0.48345 | 8.76689 | 57.58478 | 31.49107 |
| 400 | 0.46088 | 8.40583 | 55.54561 | 32.83577 |
| 600 | 0.47057 | 8.25155 | 53.60345 | 32.71285 |
| 800 | 0.47160 | 8.01712 | 52.56759 | 32.33935 |
| 1000 | 0.47176 | 7.85152 | 51.34364 | 32.18022 |

Table S2: PV parameters of the graphene CZTSSe device after different bending cycles.

| **Bending cycles (cycles)** | $V_{OC}$ **(V)** | **PCE (%)** | **FF (%)** | $J_{SC}$ **(mA cm$^{-2}$)** |
|---|---|---|---|---|
| 0 | 0.54206 | 11.42055 | 64.50012 | 32.66451 |
| 200 | 0.54035 | 11.34187 | 64.72081 | 32.43115 |
| 400 | 0.53807 | 11.20537 | 63.02537 | 33.04228 |
| 600 | 0.53529 | 11.09584 | 63.62049 | 32.58166 |
| 800 | 0.53641 | 10.95639 | 63.25412 | 32.29095 |
| 1000 | 0.53678 | 10.71023 | 62.41190 | 31.74353 |

## Calculated Thermal Stress Distribution

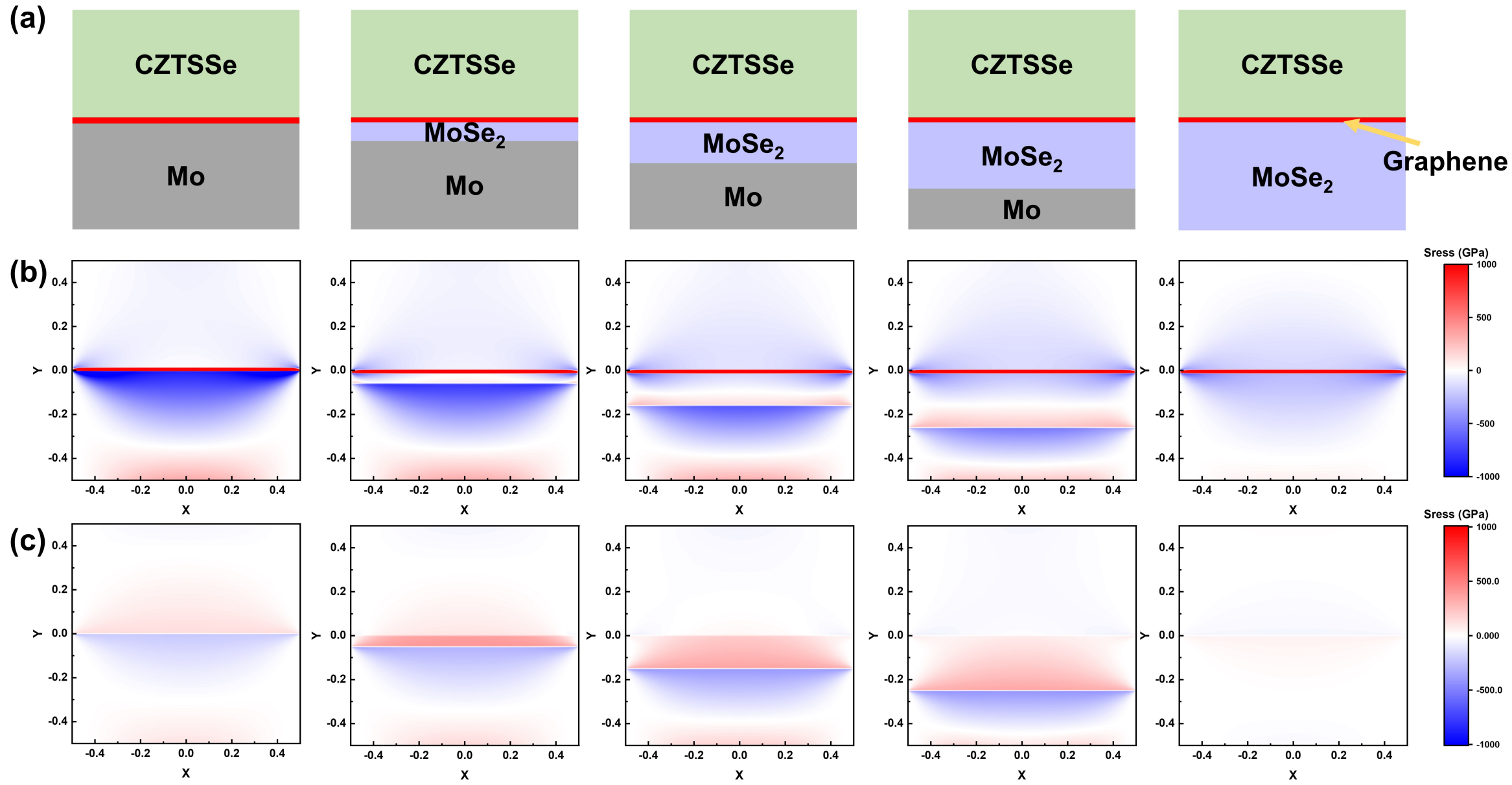


Figure S4: Calculated thermal expansion at different stages of the selenization process (stimulated with different thicknesses of $Mo(S, Se)_2$ shown in (a)). Thermal expansion of (b) with and (c) without inserted graphene.

Calculations on thermal expansion have been done in the CZTSSe devices without and with graphene inserted during the selenization process. Different stages of the selenization reaction are simulated by using different $Mo(S, Se)_2$ thicknesses at the back contact. As shown in Figure S4, the existence of $Mo(S, Se)_2$ at the beginning of the selenization reaction could induce the expansion of the Mo substrate, paving more Se diffusion paths, which might promote the formation of extremely thicker $Mo(S, Se)_2$ and cause the collapse of the back contact. Resulting of the calculation, the insertion of graphene could eliminate the thermal expansion caused by $Mo(S, Se)_2$, and keep a relatively thermal stable interface to support CZTSSe growth, even with the same thicknesses of $Mo(S, Se)_2$ at the interface. On the other hand, the thermal regulation effect from graphene will inhibit the formation of $Mo(S, Se)_2$ to some extent at the same time. Overall, this supports the flexible-specific mechanism proposed above: in metal-foil devices, thermo-mechanical effects and interfacial

reaction kinetics can reinforce each other, and graphene breaks this positive feedback loop by stabilizing the rear interface during chalcogenization.

## Defect Analysis by Admittance Spectroscopy (AS)

The defect properties of Mo foil and Mo foil/Graphene devices were investigated by using temperature-dependent AS measurements conducted from 120 K to 300 K. Figure S5 shows the AS spectra and the corresponding -f*dC/df vs. f curves derived from the AS spectra. The activation energies ($E_a$) of defects were obtained by Arrhenius fitting the $\omega_0$ values at different temperatures. The Ea values were found to be 128.1 meV and 185.9 meV for the Mo foil device, while 132.4 meV and 180.7 meV for the Mo foil/Graphene device, respectively. The lower energy is attributed to $Cu_{Na}$ and the higher one is attributed to $Cu_{Zn}$ antisite defects. The Mo/graphene device exhibits a defect-state density comparable to that of the control sample in AS measurements. This result indicates that the observed performance enhancement does not mainly originate from changes in bulk defect states, but is more closely associated with improved back-interface properties.

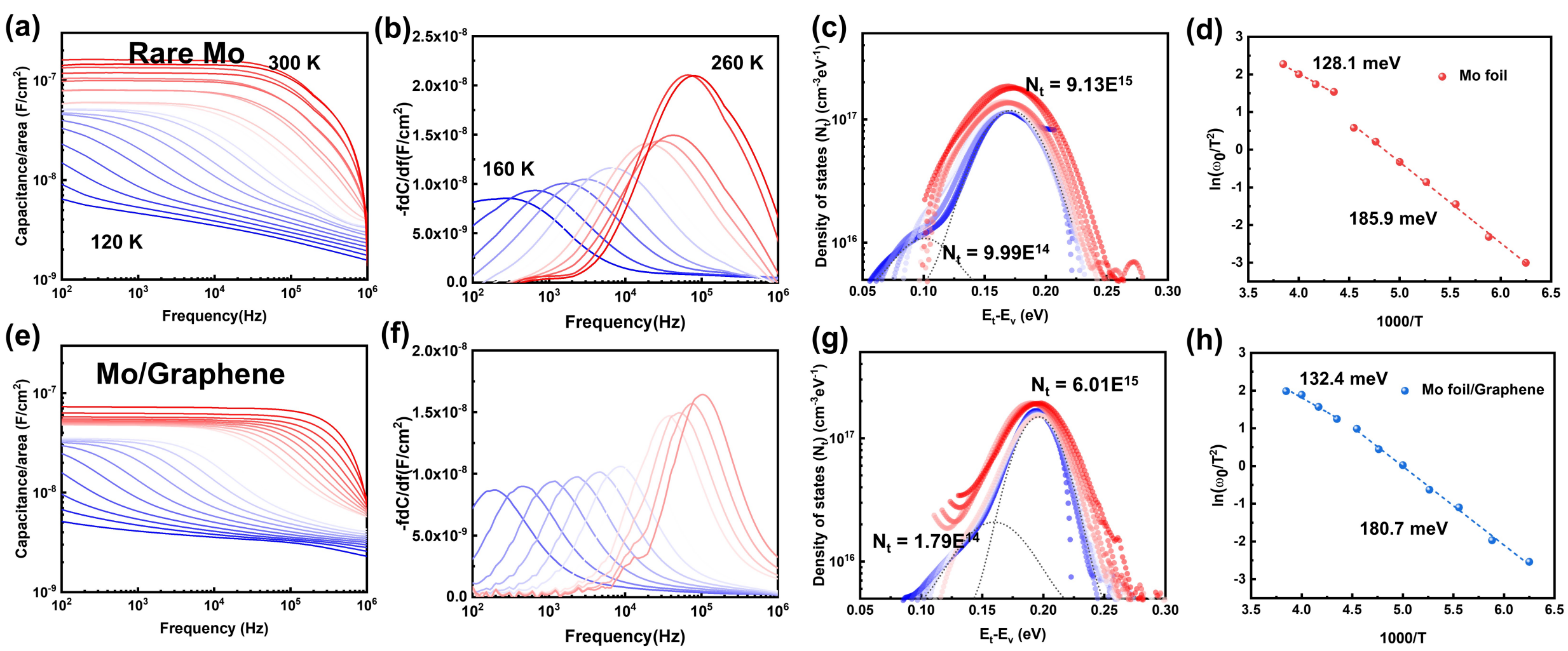


Figure S5: (a) Temperature-dependent AS spectra of CZTSSe devices with Mo foil and (b) Mo foil/Graphene substrates. (c) and (d) show the -f*dC/df f curves derived from the AS spectra. (a) and (c) present the defect activation energies obtained from the Arrhenius fits of the AS spectra for CZTSSe devices with Mo foil and Mo foil/Graphene substrates, respectively. (b) and (d) illustrate the defect state densities.

## SEM Images of As-grown Single-crystal Graphene on Copper Foil

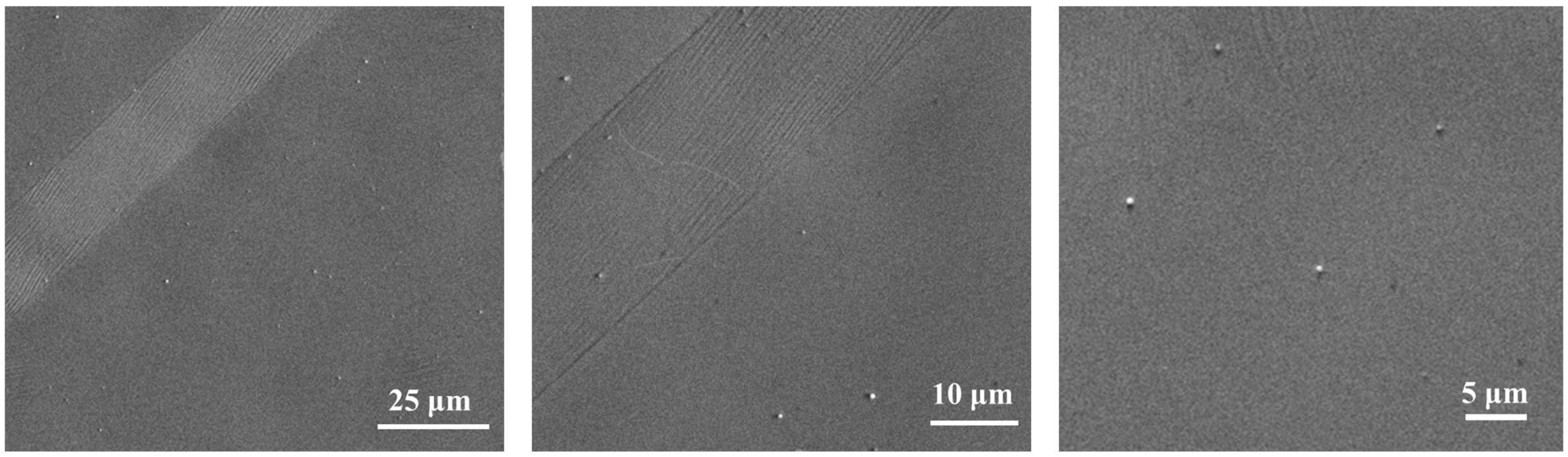


Figure S6: SEM of as-grown graphene on Cu foil from CVD.

## SEM Images of CZTSSe Layers on Mo Foil with Different Inserting Materials

A comparison between Graphene and a commonly used back contact material $Al_2O_3$ is displayed. $Al_2O_3$ was made by ALD with a thickness of 2 nm. The surface morphology of CZTSSe absorbers is shown in Figure S7. The compactness of CZTSSe was significantly improved in the sample with graphene inserted. No pins and holes can be observed from the SEM image.

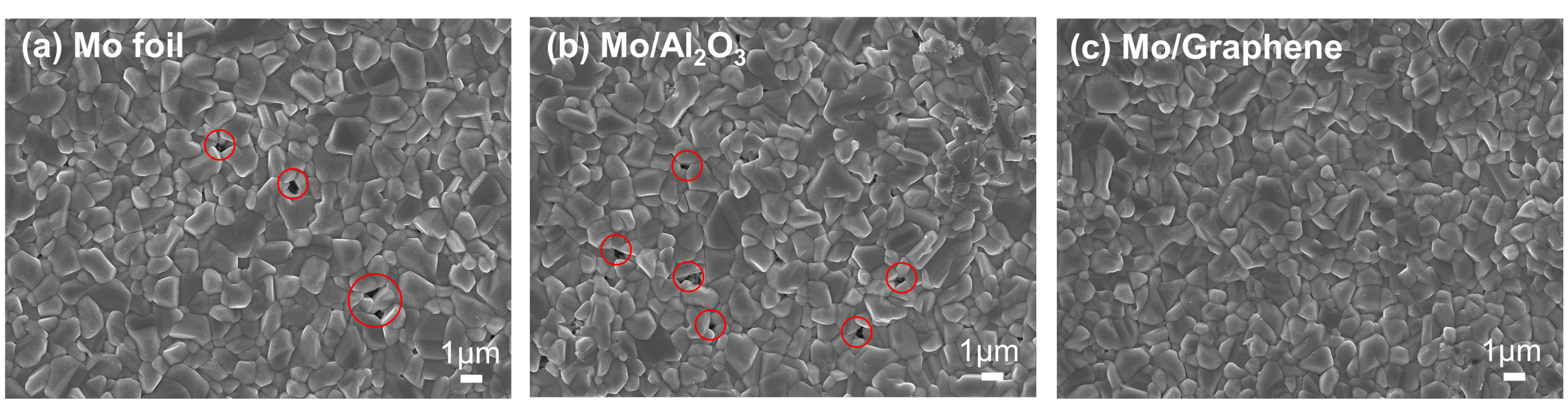


Figure S7: Surface morphology of CZTSSe on (a) bare Mo foil. (b) Mo foil/$Al_2O_3$. (c) Mo foil/Graphene.

## XRD and Raman Spectroscopy of CZTSSe Layers on Mo Foil with Different Inserting Materials

The crystal structures and phase compositions of the three samples were analyzed using X-ray diffraction (XRD) and Raman spectroscopy, as shown in Figure S8. The XRD patterns reveal that characteristic diffraction peaks of CZTSSe appear around 27.2 $^o$, 45.3 $^o$, 53.6 $^o$, and 65.9 $^o$, corresponding to the (112), (220), (312), and (040) planes of the CZTSSe structure. It is noticeable that the diffraction peaks for CZTSSe in the $Al_2O_3$ sample are lower than those in the other two samples, indicating poorer crystallinity of the absorption layer in this sample. Additionally, a comparison shows that the diffraction peaks of $Mo(S,Se)_2$ in the $Al_2O_3$ sample are slightly lower than those in the Ref and Graphene samples, while the characteristic peak of Mo (40.5 $^o$) is significantly higher than in the other two samples. This suggests that the Mo foil/$Al_2O_3$/CZTSSe substrate structure suffers from severe stress release issues, leading to reduced peak intensity for CZTSSe and $Mo(S,Se)_2$ and increased intensity for Mo. Figure S8(b) displays the Raman spectra of the absorption layers for the three substrates. Characteristic peaks for CZTSSe are observed at 170 $cm^{-1}$, 199 $cm^{-1}$, and 237 $cm^{-1}$ in three samples, with the peak intensities in the $Al_2O_3$ sample being lower than in the other two samples, consistent with the XRD results. Furthermore, the peak at 199 $cm^{-1}$ in the graphene sample is slightly shifted to the right compared to the Ref sample, indicating an increase in the unit cell size. Compared to isolating ultra-thin $Al_2O_3$, graphene is a better choice for rear interface of CZTSSe solar cells.

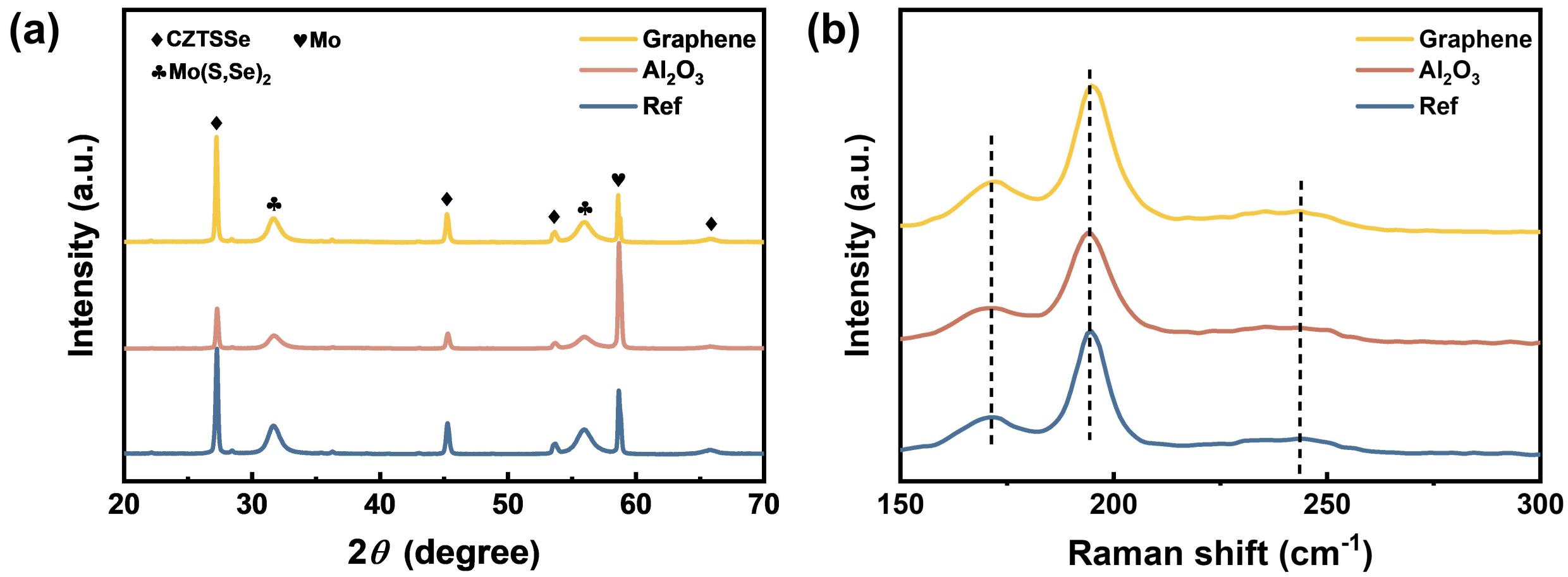


Figure S8: (a) XRD of CZTSSe on Mo foil, Mo foil/$Al_2O_3$, and Mo foil/Graphene. (b) Raman spectrum.

## PV Performance

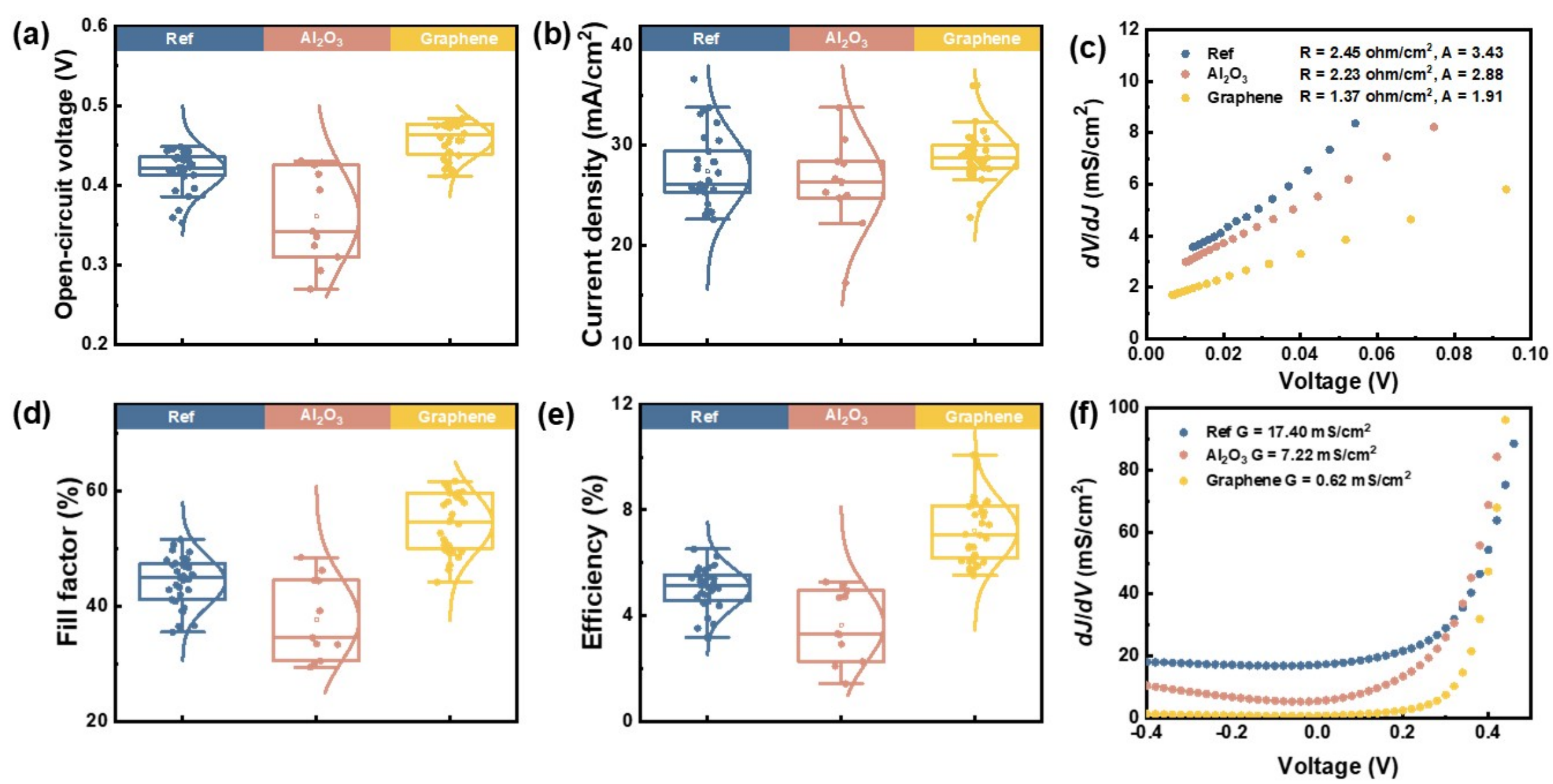


Figure S9: Statistical data of a) Voc, b) Jsc, c) FF, and d) Overall solar conversion efficiency of the devices (PCE).

## Calculated diode parameters from Champion cells

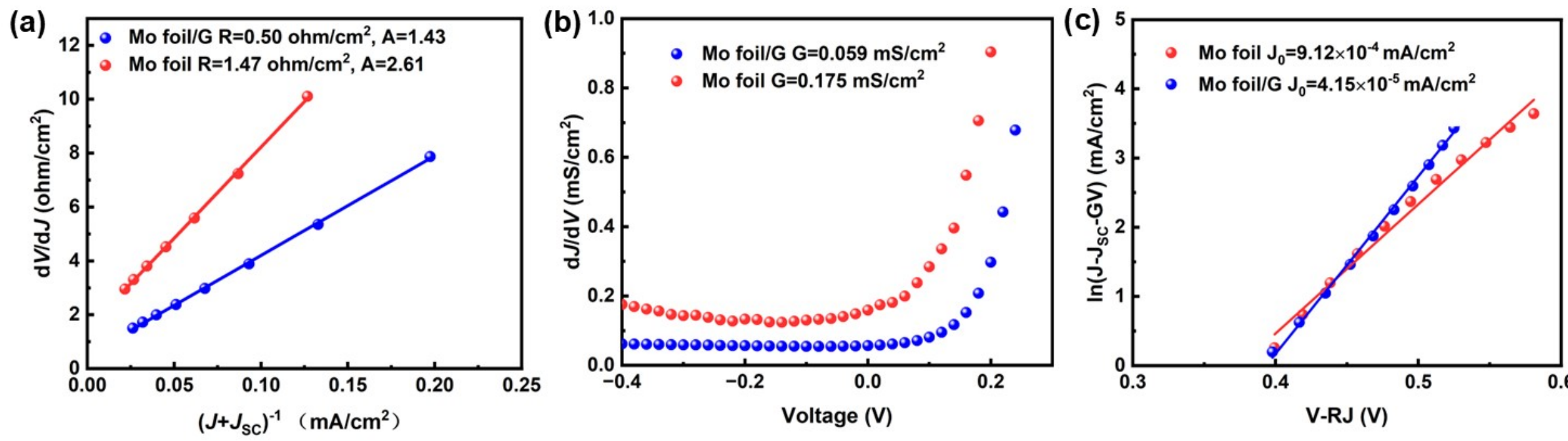


Figure S10: Calculated diode parameters. (a) the series resistance Rs and diode quality factor A, and (b) the parallel conductance $G_{sh}$, were calculated. Blue is the Mo foil sample, red is the one with $Al_2O_3$ inserted, and yellow is the Mo foil/Graphene sample.

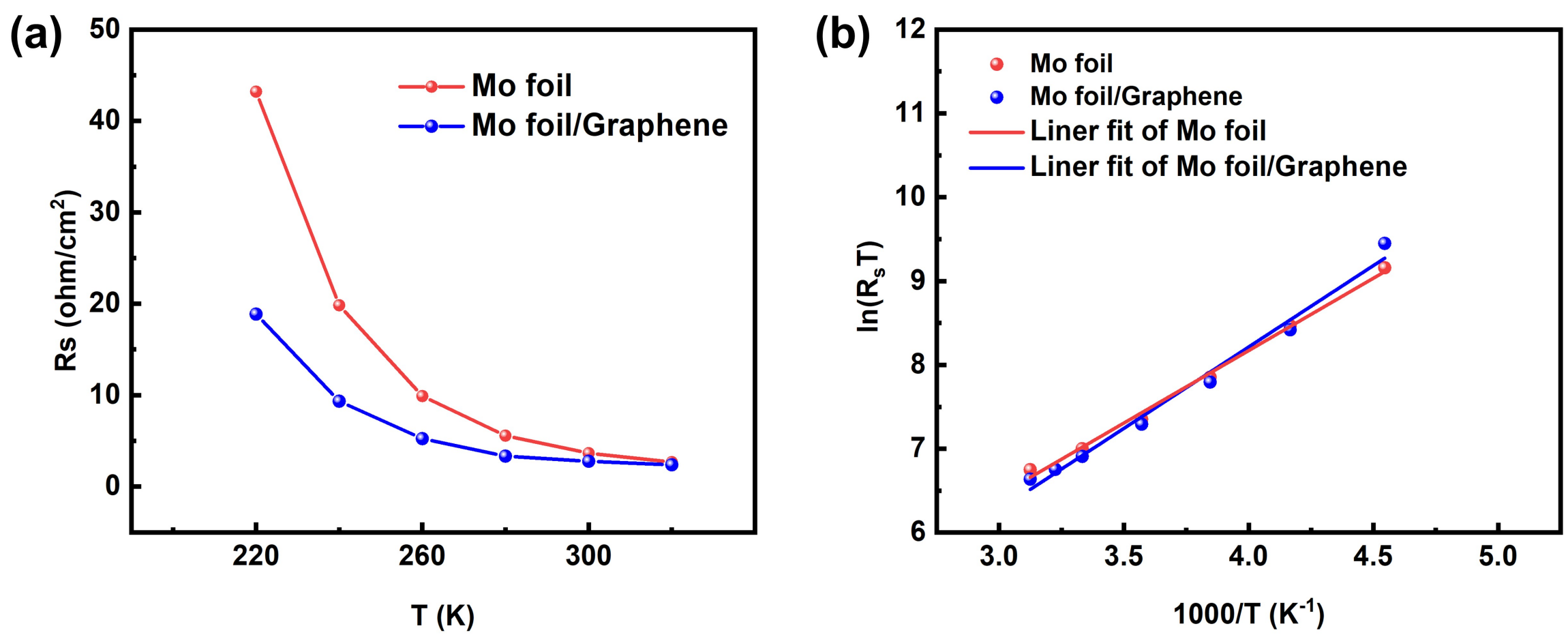


Figure S11: (a) $R_S$ derived from temperature-dependent dark J-V curves. (b) Plot of Ln(RsT) vs. 1000/T.

The back-contact barrier height was extracted from temperature-dependent dark $J$–$V$ measurements over 240–330 K. Within this range, the series resistance derived from Equation 1,

$$\frac{\mathrm{d}V}{\mathrm{d}J} = R_\mathrm{s} + \frac{n k_\mathrm{B} T}{q(J - G_\mathrm{s} V)},$$

increases monotonically with decreasing temperature, consistent with thermally activated transport across the rear interface. Moreover, the resistance exhibits near-linear Arrhenius behavior in this temperature range, enabling reliable barrier-height extraction using Equation 2:

$$R_{\mathrm{BC}} = \frac{K}{qA^{*}T} \exp\left(\frac{\Phi_{\mathrm{B}}}{k_{\mathrm{B}}T}\right).$$

The equivalent circuit comprises a main solar-cell diode ($D_{\mathrm{SC}}$), a background series resistance ($R_0$) arising from the upper layers and CZTSSe bulk, and a back-contact diode ($D_{\mathrm{BC}}$) connected in reverse series with $D_{\mathrm{SC}}$. Under forward bias, $D_{\mathrm{BC}}$ is reverse-biased; therefore, its reverse saturation current governs the back-contact contribution to the total series resistance.

A thick $MoSe_2$ layer induces pronounced band bending and a hole-transport barrier, $\Phi_{\mathrm{B}}$, at the CZTSSe/Mo interface. The temperature dependence of $D_{\mathrm{BC}}$ is therefore described by the thermionic-emission model in Equation 2. Because $R_0$ is assumed to vary only weakly with temperature, the strong temperature dependence of the total series resistance is primarily attributed to $R_{\mathrm{BC}}$. Accordingly, $\Phi_{\mathrm{B}}$ can be extracted from the slope of $\ln(R_{\mathrm{BC}}T)$ versus $1/T$.

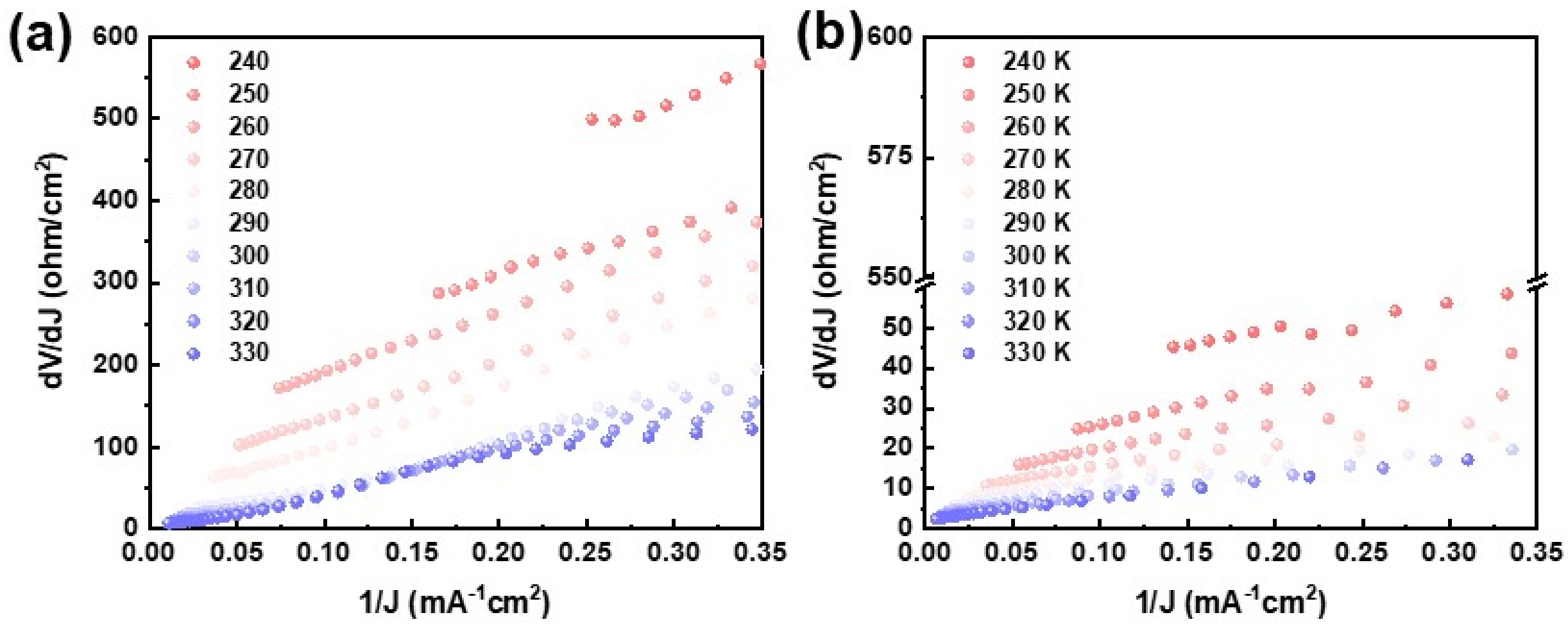


Figure S12: Plot of dV/dJ vs. 1/J. for (a) Mo foil device and (b) Mo foil/Graphene device.
