# Supplementary material for: Graphene‐Engineered Rear Interfaces Enable Efficient Flexible CZTSSe Solar Cells on Metal Foils
Source: Adv Sci (Weinh). 2026 Aug 17:e77181. Online ahead of print. doi: 10.1002/advs.77181 (PMC13479858; doi:10.1002/advs.77181)
Supplement: Supplementary file 1 [file ADVS-9999-e77181-s001.pdf]

Supporting Information Available

Bending Test of flexible cells

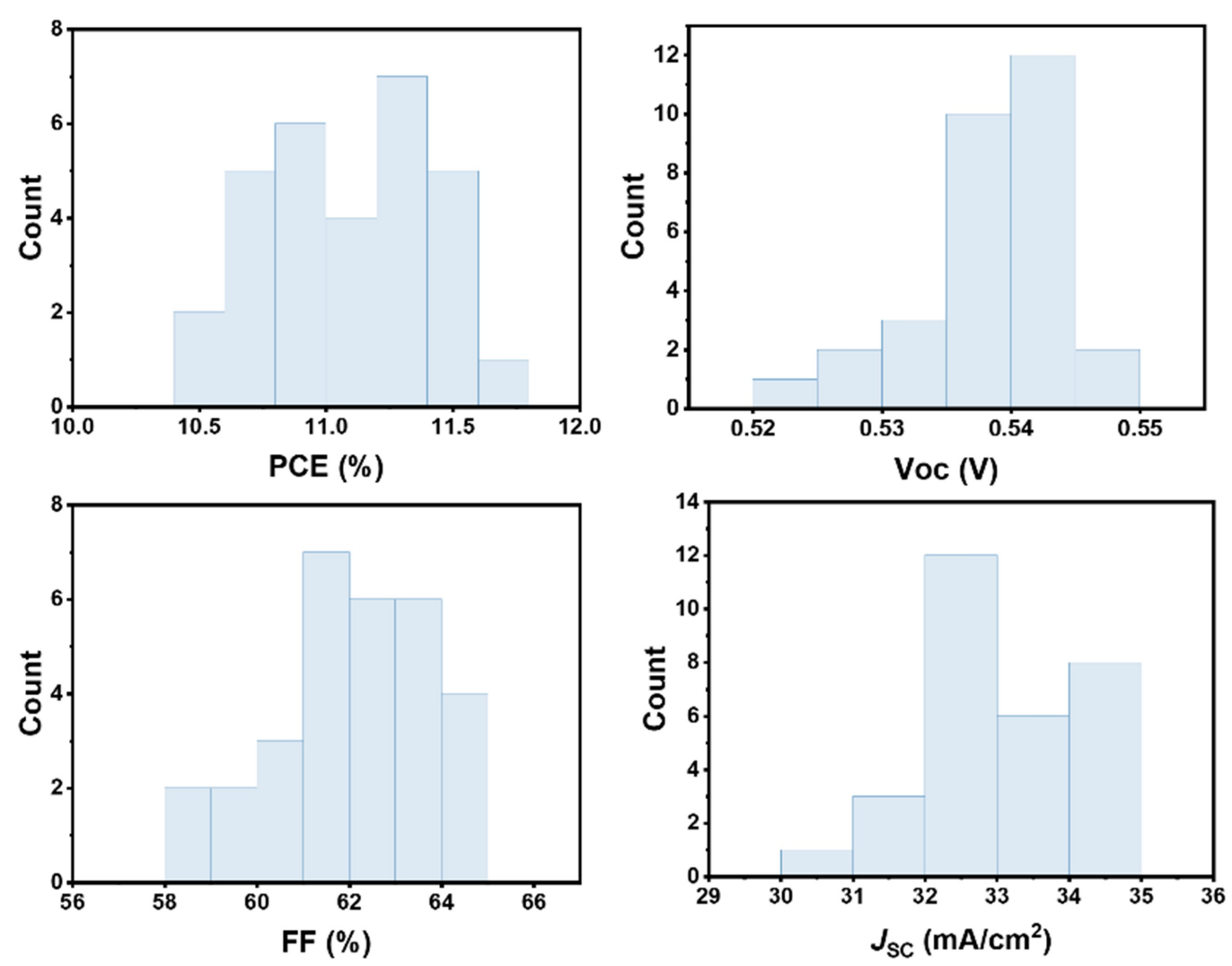

Figure S1: The photovoltaic properties distribution of Mo foil/Graphene devices.

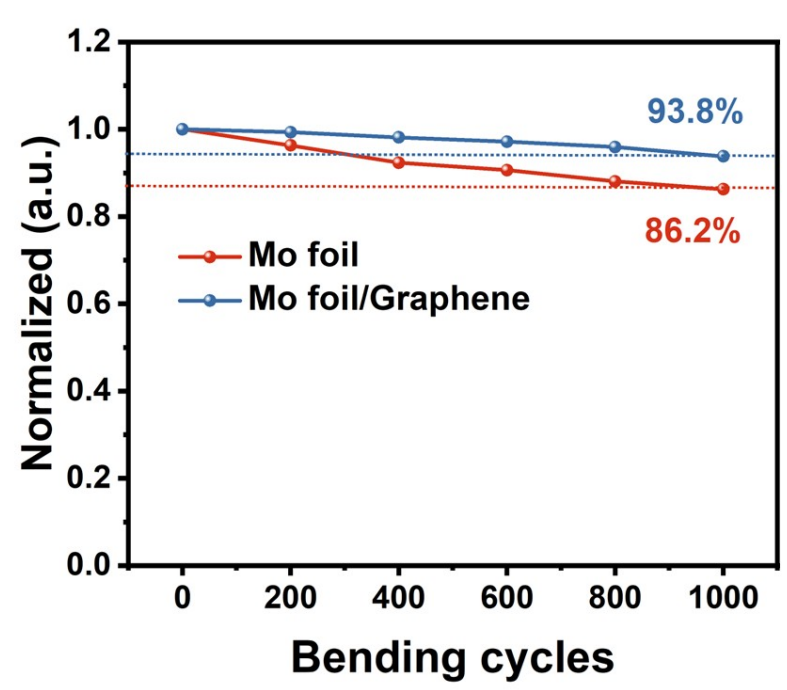

Figure S2: The normalized PCEs of CZTSSe devices as a function of bending cycles.

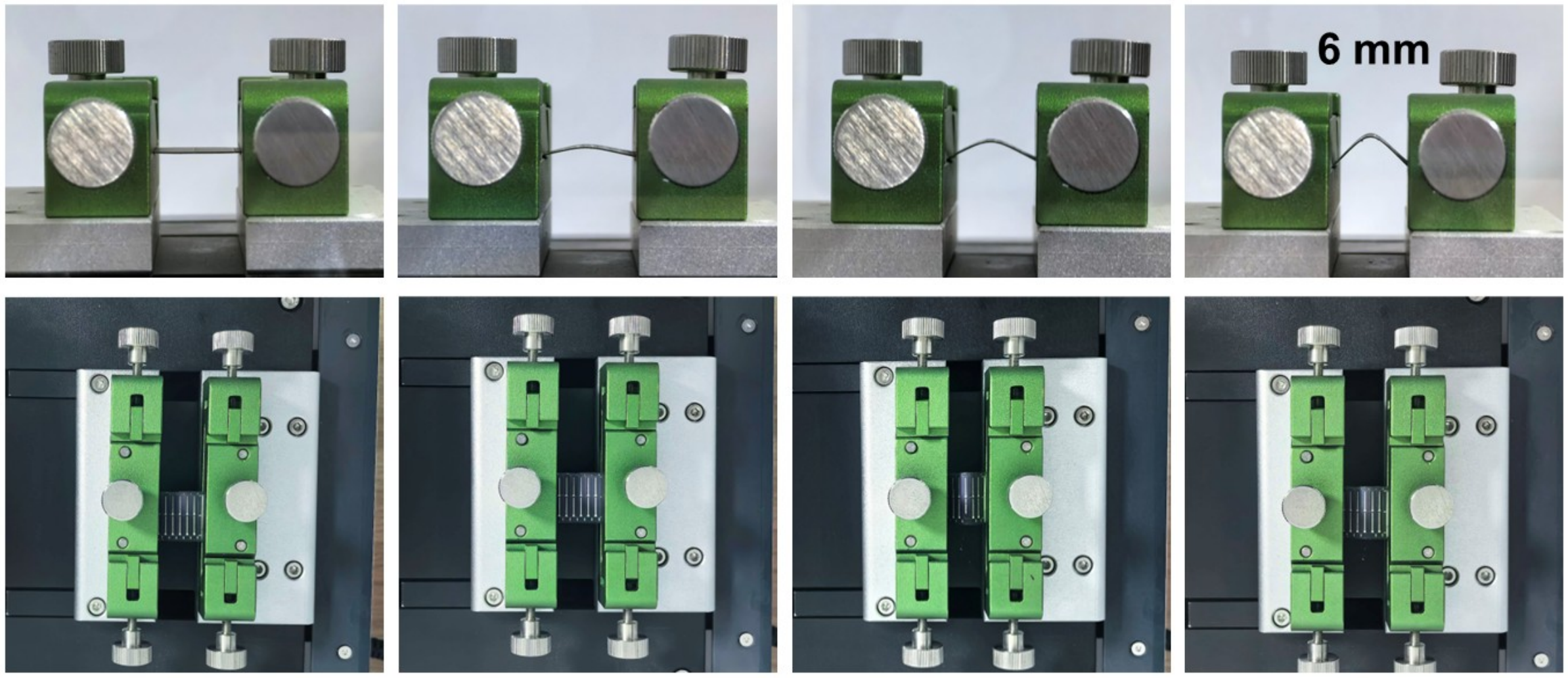

Figure S3: Bending test of flexible CZTSSe solar cells.

Table S1: Photovoltaic parameters of the Mo CZTSSe device after different bending cycles.

**Bending cycles (cycles)**	V_{OC} **(V)**	**PCE (%)**	**FF (%)**	J_{SC} **(mA cm^{-2})**
0	0.48666	9.10489	60.01336	31.17436
200	0.48345	8.76689	57.58478	31.49107
400	0.46088	8.40583	55.54561	32.83577
600	0.47057	8.25155	53.60345	32.71285
800	0.47160	8.01712	52.56759	32.33935
1000	0.47176	7.85152	51.34364	32.18022

Table S2: PV parameters of the graphene CZTSSe device after different bending cycles.

**Bending cycles (cycles)**	V_{OC} **(V)**	**PCE (%)**	**FF (%)**	J_{SC} **(mA cm^{-2})**
0	0.54206	11.42055	64.50012	32.66451
200	0.54035	11.34187	64.72081	32.43115
400	0.53807	11.20537	63.02537	33.04228
600	0.53529	11.09584	63.62049	32.58166
800	0.53641	10.95639	63.25412	32.29095
1000	0.53678	10.71023	62.41190	31.74353

Calculated Thermal Stress Distribution

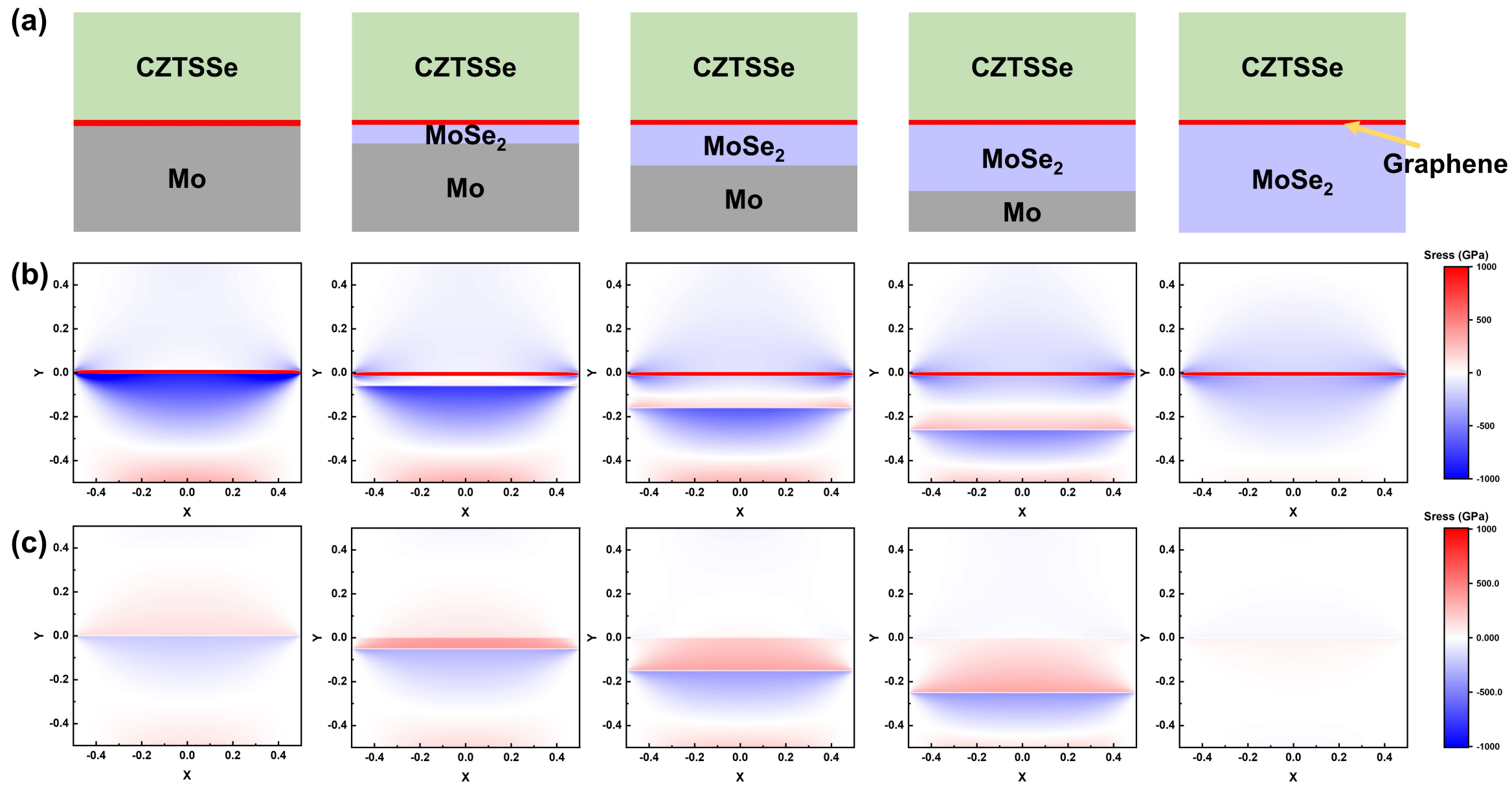

Figure S4: Calculated thermal expansion at different stages of the selenization process (stimulated with different thicknesses of Mo(S, Se)$_2$ shown in (a)). Thermal expansion of (b) with and (c) without inserted graphene.

Calculations on thermal expansion have been done in the CZTSSe devices without and with graphene inserted during the selenization process. Different stages of the selenization reaction are simulated by using different Mo(S, Se)$_2$ thicknesses at the back contact. As shown in Figure S4, the existence of Mo(S, Se)$_2$ at the beginning of the selenization reaction could induce the expansion of the Mo substrate, paving more Se diffusion paths, which might promote the formation of extremely thicker Mo(S, Se)$_2$ and cause the collapse of the back contact. Resulting of the calculation, the insertion of graphene could eliminate the thermal expansion caused by Mo(S, Se)$_2$, and keep a relatively thermal stable interface to support CZTSSe growth, even with the same thicknesses of Mo(S, Se)$_2$ at the interface. On the other hand, the thermal regulation effect from graphene will inhibit the formation of Mo(S, Se)$_2$ to some extent at the same time. Overall, this supports the flexible-specific mechanism proposed above: in metal-foil devices, thermo-mechanical effects and interfacial

reaction kinetics can reinforce each other, and graphene breaks this positive feedback loop by stabilizing the rear interface during chalcogenization.

Defect Analysis by Admittance Spectroscopy (AS)

The defect properties of Mo foil and Mo foil/Graphene devices were investigated by using temperature-dependent AS measurements conducted from 120 K to 300 K. Figure S5 shows the AS spectra and the corresponding -f*dC/df vs. f curves derived from the AS spectra. The activation energies (E_a) of defects were obtained by Arrhenius fitting the ω_0 values at different temperatures. The Ea values were found to be 128.1 meV and 185.9 meV for the Mo foil device, while 132.4 meV and 180.7 meV for the Mo foil/Graphene device, respectively. The lower energy is attributed to Cu_{Na} and the higher one is attributed to Cu_{Zn} antisite defects. The Mo/graphene device exhibits a defect-state density comparable to that of the control sample in AS measurements. This result indicates that the observed performance enhancement does not mainly originate from changes in bulk defect states, but is more closely associated with improved back-interface properties.

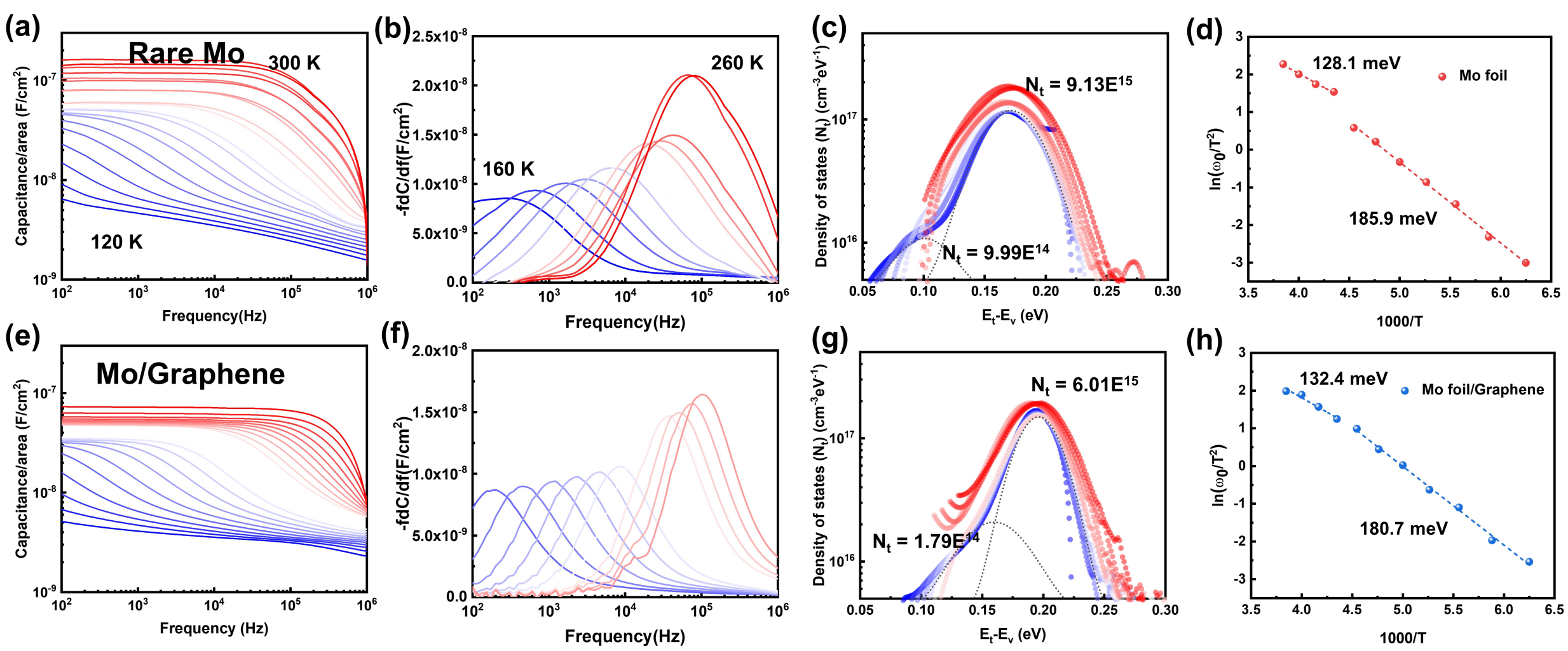

Figure S5: (a) Temperature-dependent AS spectra of CZTSSe devices with Mo foil and (b) Mo foil/Graphene substrates. (c) and (d) show the -f*dC/df f curves derived from the AS spectra. (a) and (c) present the defect activation energies obtained from the Arrhenius fits of the AS spectra for CZTSSe devices with Mo foil and Mo foil/Graphene substrates, respectively. (b) and (d) illustrate the defect state densities.

SEM Images of As-grown Single-crystal Graphene on Copper Foil

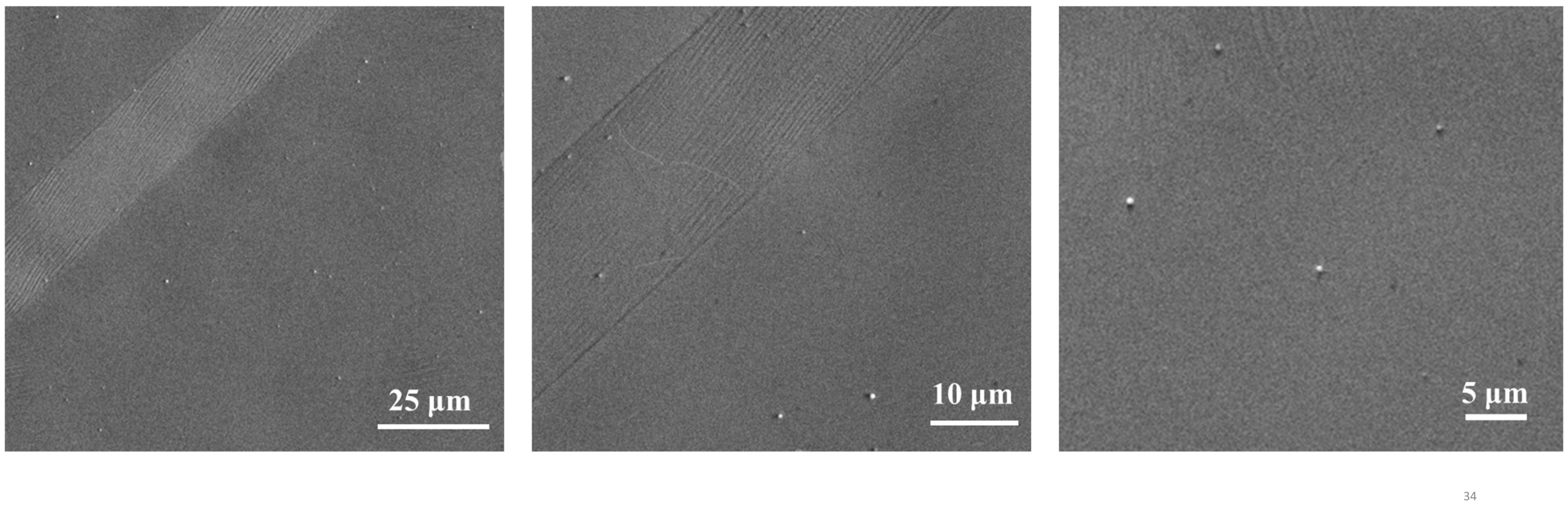

Figure S6: SEM of as-grown graphene on Cu foil from CVD.

SEM Images of CZTSSe Layers on Mo Foil with Different Inserting Materials

A comparison between Graphene and a commonly used back contact material Al_2O_3 is displayed. Al_2O_3 was made by ALD with a thickness of 2 nm. The surface morphology of CZTSSe absorbers is shown in Figure S7. The compactness of CZTSSe was significantly improved in the sample with graphene inserted. No pins and holes can be observed from the SEM image.

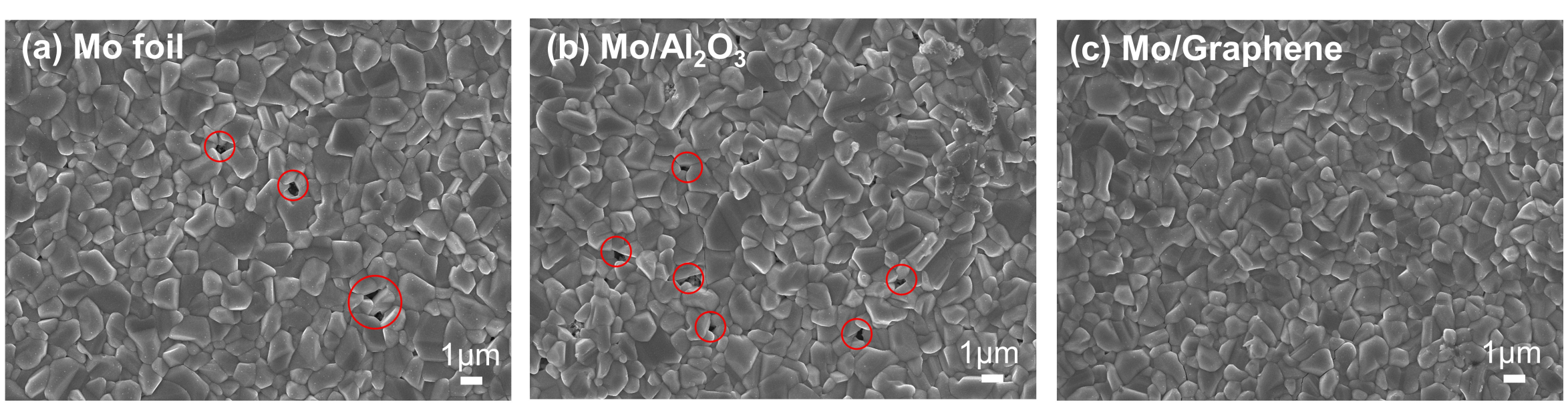

Figure S7: Surface morphology of CZTSSe on (a) bare Mo foil. (b) Mo foil/Al_2O_3. (c) Mo foil/Graphene.

XRD and Raman Spectroscopy of CZTSSe Layers on Mo Foil with Different Inserting Materials

The crystal structures and phase compositions of the three samples were analyzed using X-ray diffraction (XRD) and Raman spectroscopy, as shown in Figure S8. The XRD patterns reveal that characteristic diffraction peaks of CZTSSe appear around 27.2 ^{o}, 45.3 ^{o}, 53.6 ^{o}, and 65.9 ^{o}, corresponding to the (112), (220), (312), and (040) planes of the CZTSSe structure. It is noticeable that the diffraction peaks for CZTSSe in the Al_2O_3 sample are lower than those in the other two samples, indicating poorer crystallinity of the absorption layer in this sample. Additionally, a comparison shows that the diffraction peaks of $Mo(S,Se)_2$ in the Al_2O_3 sample are slightly lower than those in the Ref and Graphene samples, while the characteristic peak of Mo (40.5 ^{o}) is significantly higher than in the other two samples. This suggests that the Mo foil/Al_2O_3/CZTSSe substrate structure suffers from severe stress release issues, leading to reduced peak intensity for CZTSSe and $Mo(S,Se)_2$ and increased intensity for Mo. Figure S8(b) displays the Raman spectra of the absorption layers for the three substrates. Characteristic peaks for CZTSSe are observed at 170 cm^{-1}, 199 cm^{-1}, and 237 cm^{-1} in three samples, with the peak intensities in the Al_2O_3 sample being lower than in the other two samples, consistent with the XRD results. Furthermore, the peak at 199 cm^{-1} in the graphene sample is slightly shifted to the right compared to the Ref sample, indicating an increase in the unit cell size. Compared to isolating ultra-thin Al_2O_3, graphene is a better choice for rear interface of CZTSSe solar cells.

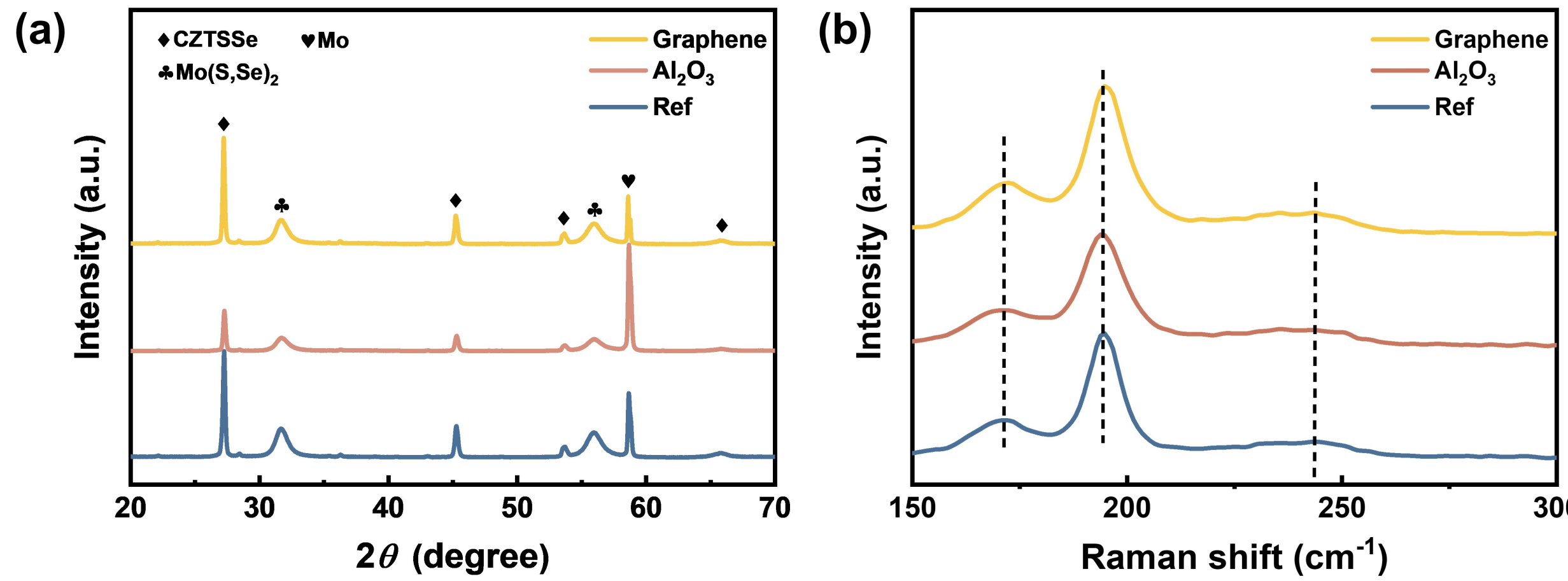

Figure S8: (a) XRD of CZTSSe on Mo foil, Mo foil/Al_2O_3, and Mo foil/Graphene. (b) Raman spectrum.

PV Performance

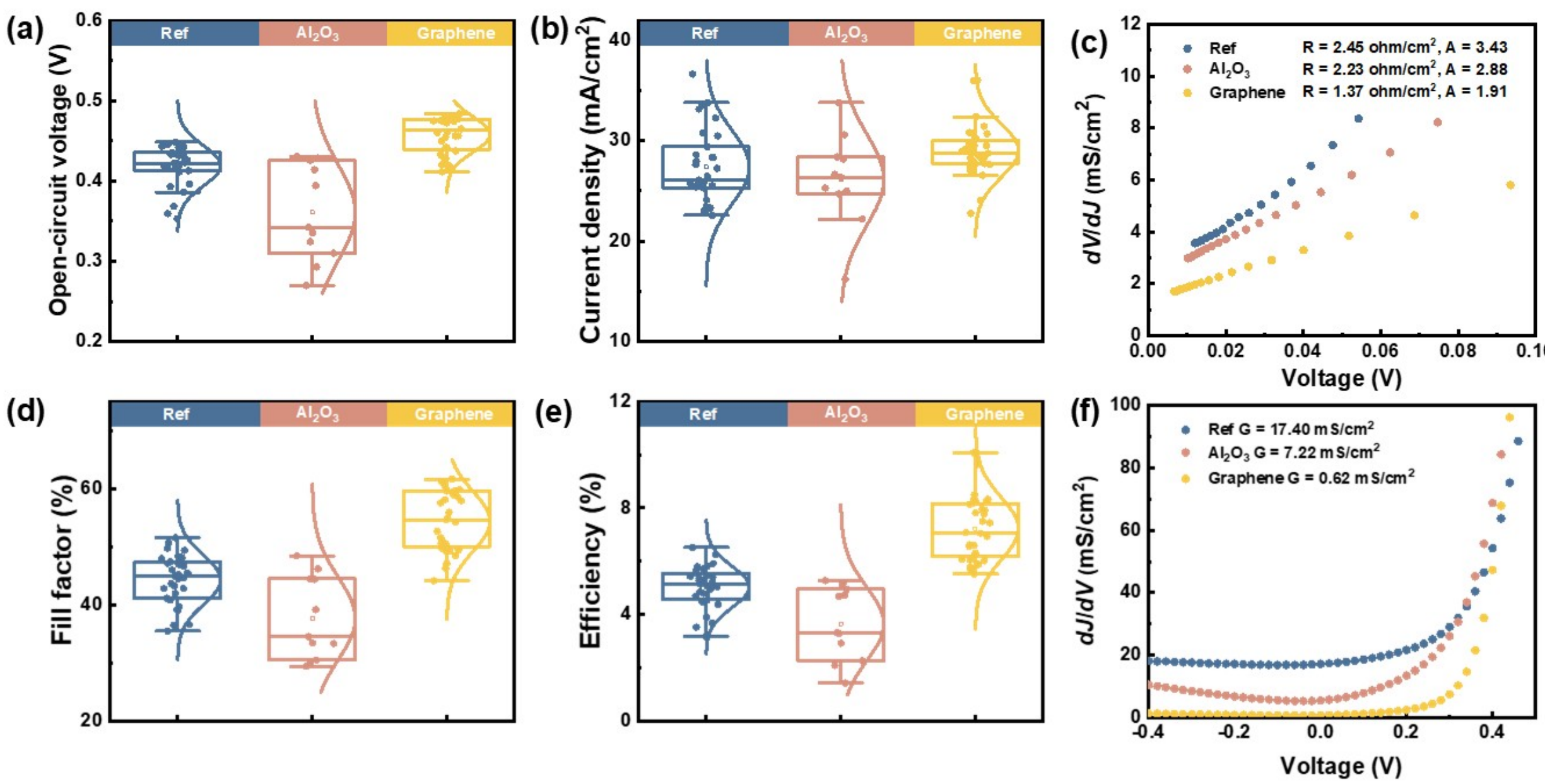

Figure S9: Statistical data of a) Voc, b) Jsc, c) FF, and d) Overall solar conversion efficiency of the devices (PCE).

Calculated diode parameters from Champion cells

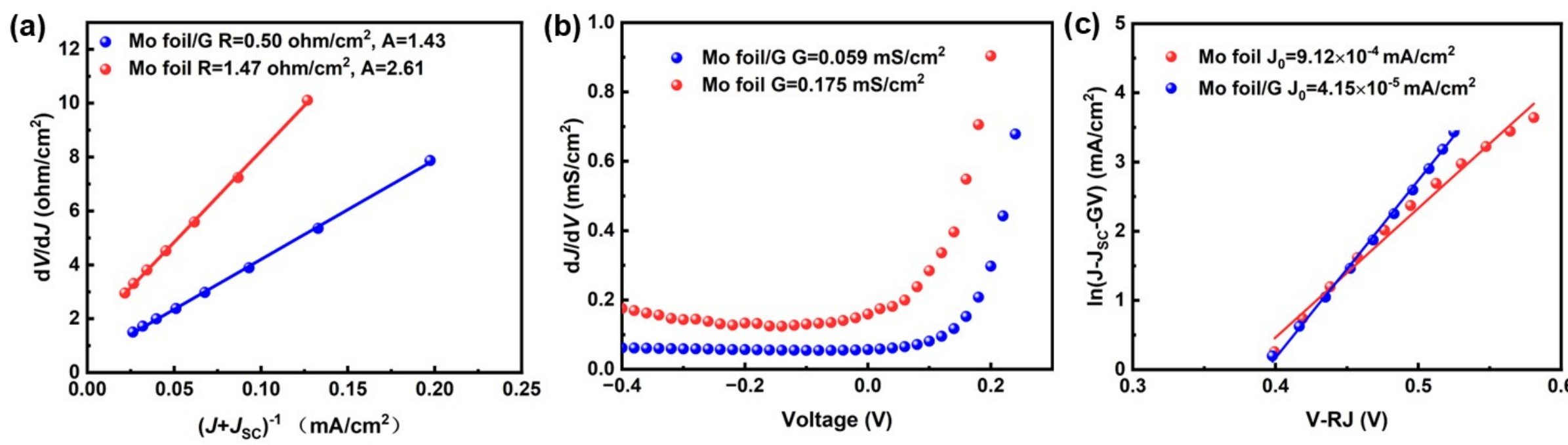

Figure S10: Calculated diode parameters. (a) the series resistance Rs and diode quality factor A, and (b) the parallel conductance G_{sh}, were calculated. Blue is the Mo foil sample, red is the one with Al_2O_3 inserted, and yellow is the Mo foil/Graphene sample.

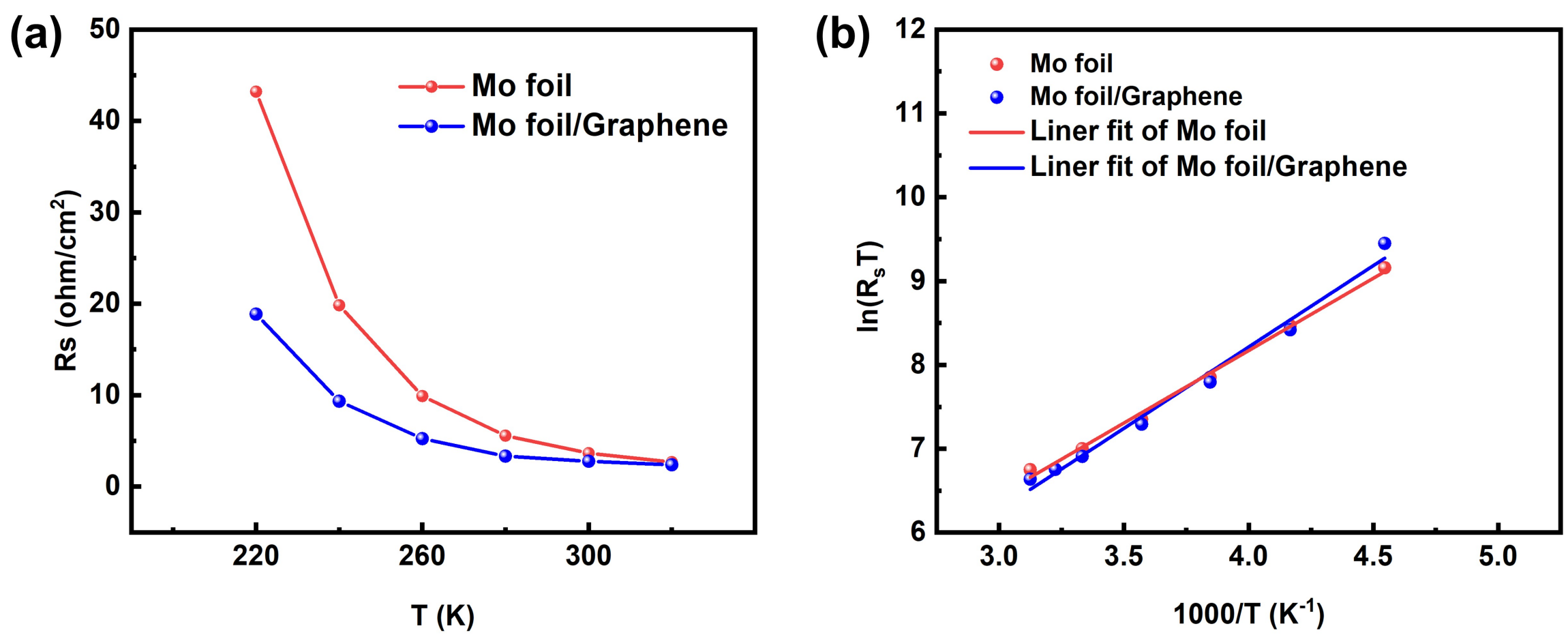

Figure S11: (a) R_S derived from temperature-dependent dark J-V curves. (b) Plot of Ln(RsT) vs. 1000/T.

The back-contact barrier height was extracted from temperature-dependent dark J–V measurements over 240–330 K. Within this range, the series resistance derived from Equation 1,

$$\frac{\mathrm{d}V}{\mathrm{d}J} = R_\mathrm{s} + \frac{nk_\mathrm{B}T}{q(J - G_\mathrm{s}V)},$$

increases monotonically with decreasing temperature, consistent with thermally activated transport across the rear interface. Moreover, the resistance exhibits near-linear Arrhenius behavior in this temperature range, enabling reliable barrier-height extraction using Equation 2:

$$R_{\mathrm{BC}} = \frac{K}{qA^{*}T}\exp\left(\frac{\Phi_{\mathrm{B}}}{k_{\mathrm{B}}T}\right).$$

The equivalent circuit comprises a main solar-cell diode (D_{SC}), a background series resistance (R_0) arising from the upper layers and CZTSSe bulk, and a back-contact diode (D_{BC}) connected in reverse series with D_{SC}. Under forward bias, D_{BC} is reverse-biased; therefore, its reverse saturation current governs the back-contact contribution to the total series resistance.

A thick $MoSe_2$ layer induces pronounced band bending and a hole-transport barrier, Φ_{B}, at the CZTSSe/Mo interface. The temperature dependence of D_{BC} is therefore described by the thermionic-emission model in Equation 2. Because R_0 is assumed to vary only weakly with temperature, the strong temperature dependence of the total series resistance is primarily attributed to R_{BC}. Accordingly, Φ_{B} can be extracted from the slope of $\ln(R_{\mathrm{BC}}T)$ versus $1/T$.

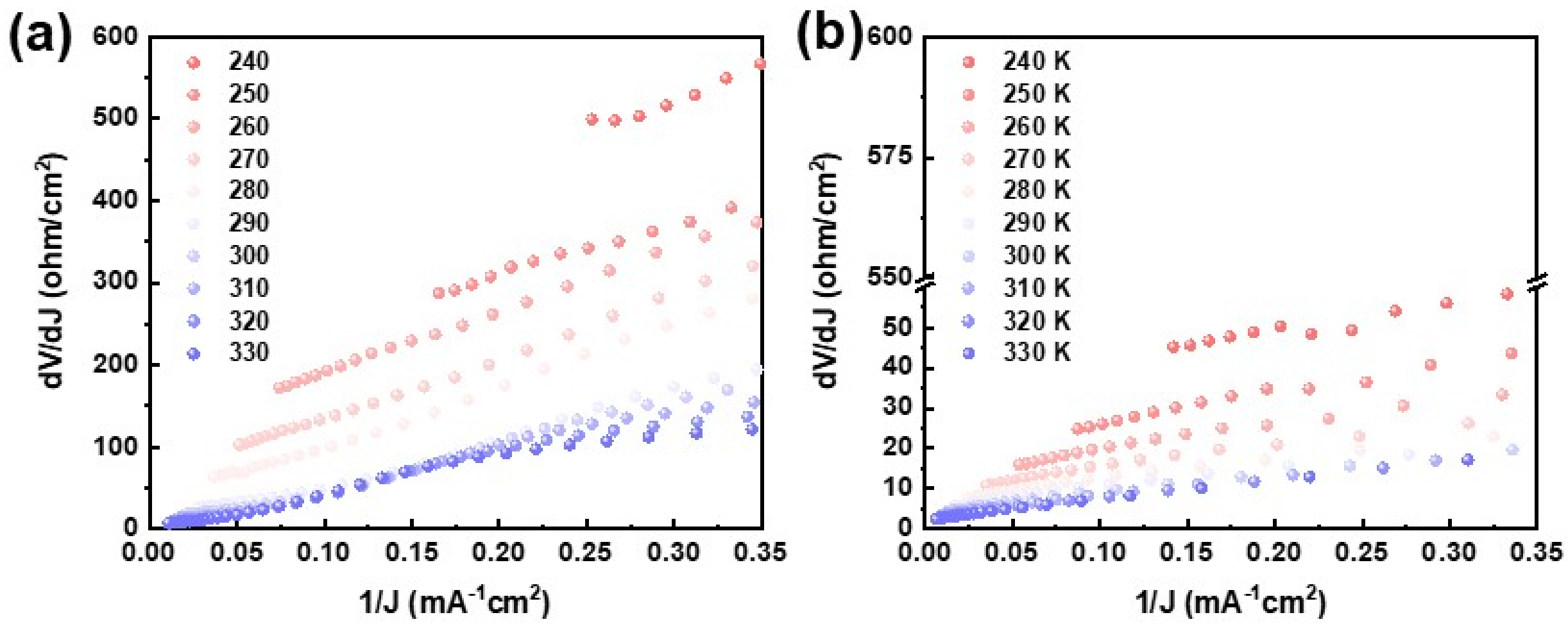

Figure S12: Plot of dV/dJ vs. 1/J. for (a) Mo foil device and (b) Mo foil/Graphene device.
